# Web-based cardiovascular disease risk prediction using machine learning

**DOI:** 10.3389/frai.2026.1690664

**Published:** 2026-02-13

**Authors:** Suraiya Akhter, John H. Miller

**Affiliations:** 1 School of Business and Technology, Emporia State University, Emporia, KS, United States; 2 School of Engineering and Applied Sciences, Washington State University, Richland, WA, United States

**Keywords:** cardiovascular disease risk prediction, feature selection, machine learning, SHAP, web application

## Abstract

Cardiovascular disease (CVD) remains the foremost contributor to global illness and death, underscoring the critical need for effective tools that can predict risk at early stages to support preventive care and timely clinical decisions. With the growing complexity of healthcare data, machine learning has shown considerable promise in extracting insights that enhance medical decision-making. Nonetheless, the effectiveness and clarity of machine learning models largely rely on the relevance and quality of input features. In this work, we explored and compared four feature-selection strategies—Pearson correlation + Chi-squared test, Alternating Decision Tree (ADT)-based scoring, Cross-Validated Feature Evaluation (CVFE), and Hypergraph-Based Feature Evaluation (HFE)—to identify the most predictive factors for CVD risk. Our analysis utilized data from the National Health and Nutrition Examination Survey (NHANES), administered by the National Center for Health Statistics under the Centers for Disease Control and Prevention (CDC), encompassing demographic, clinical, laboratory, and survey data collected across the U.S. from August 2021 through August 2023. Distinct sets of features obtained through these selection techniques were used to develop random forest (RF), support vector machine (SVM), and eXtreme Gradient Boosting (XGBoost) models, which were then assessed for predictive effectiveness. To improve clarity and understanding of model decision-making, SHapley Additive exPlanations (SHAP) was used to interpret feature contributions in the top-performing model. Among the evaluated methods, the HFE approach combined with SVM achieved the highest overall accuracy (82.84%) and AUC (0.9027), outperforming both classical and alternative strategies. The most influential predictors included age, total cholesterol, history of high blood pressure, use of cholesterol-lowering medication, recent prescription medication use, lifetime smoking history, family income-to-poverty ratio, gender, educational attainment, and red cell distribution width. The web application, accessible at https://shiny.tricities.wsu.edu/cvdr-prediction/, presents predictive results, probability scores, and SHAP plots generated from the model trained using the feature set selected by the hypergraph-based approach. This study highlights the importance of strategic feature selection in refining predictive accuracy and interpretability, offering a practical data-driven approach that could aid clinicians in evaluating cardiovascular risk and tailoring preventive care.

## Introduction

Globally, cardiovascular diseases (CVDs) remain the primary cause of death, responsible for around 17.9 million fatalities each year, which equates to nearly one-third of all deaths worldwide (World Health Organization, 2017). This broad category includes multiple heart and circulatory system disorders such as coronary artery disease, stroke, peripheral artery disease, rheumatic heart conditions, and congenital cardiovascular defects (Ogunpola et al., 2024; Mousa et al., 2014). Among these, coronary artery disease constitutes the majority, accounting for approximately 64% of CVD occurrences (Ogunpola et al., 2024). These statistics underscore the urgent need for effective early detection strategies and preventive interventions to reduce the global burden of CVD. CVD risk arises from a multifaceted interaction between modifiable and non-modifiable factors. Lifestyle related factors including elevated cholesterol, diabetes, obesity, tobacco use, and physical inactivity are among the most influential modifiable contributors (Sen, 2017; Yazdani et al., 2021). In contrast, non-modifiable determinants include age, biological sex, and racial or ethnic background (Guarneros-Nolasco et al., 2021; Mandava, 2024; Pu et al., 2012; Hossen et al., 2021). The widespread adoption of unhealthy behaviors in today’s society has intensified these risks (Pouriyeh et al., 2017; Ambekar and Phalnikar, 2018). Therefore, identifying high-risk individuals with precision and at an early stage is essential to enable preventive actions, slow disease progression, and decrease mortality rates.

Several traditional clinical risk assessment tools, such as the Framingham Risk Score, SCORE charts, and the REGICOR model, have been widely used to estimate cardiovascular risk (Gil-Guillen et al., 2007; Khandoker et al., 2019; Assmann et al., 2002). However, these approaches often rely on a limited set of predictors and assume linear relationships, potentially oversimplifying the complex mechanisms underlying CVD. In addition, their performance may vary across populations, limiting generalizability. To overcome these limitations, machine learning has emerged as a powerful alternative for cardiovascular risk prediction. Machine learning methods can model nonlinear and high-order interactions among heterogeneous variables, offering greater flexibility and predictive accuracy than traditional statistical techniques (Patel et al., 2015; Solanki and Barot, 2019; Kiran et al., 2022). Commonly used machine learning algorithms include decision trees, support vector machines, *k*-nearest neighbors, random forests, gradient boosting methods, XGBoost, and deep learning architectures such as convolutional neural networks (Ogunpola et al., 2024; Mandava, 2024; Azmi et al., 2022; Rahim et al., 2021; Rubini et al., 2021; Elsayed and Syed, 2017; DeGroat et al., 2024).

Numerous machine learning-based studies have demonstrated improved performance in predicting CVD risk using both clinical and population-level datasets (Azmi et al., 2022; Rahim et al., 2021; Rubini et al., 2021; Elsayed and Syed, 2017; DeGroat et al., 2024; Peng et al., 2023; Shishehbori and Awan, 2024; Mansoori et al., 2024; van Os et al., 2023; Neumann et al., 2022; Wallisch et al., 2021). Meta-analyses indicate that ensemble and neural-network models often outperform conventional statistical approaches (Cai et al., 2024; Krittanawong et al., 2020). However, social and behavioral determinants remain underrepresented in many models (Zhao et al., 2021). Several explainable-AI frameworks combining machine learning with SHAP have revealed key predictors—blood pressure, lipids, glycated hemoglobin, inflammatory markers, and smoking status—that drive model decisions (Peng et al., 2023; Lundberg et al., 2020). Large-scale population datasets such as NHANES have been widely used to model cardiovascular risk across diverse socioeconomic and lifestyle profiles (Peng et al., 2023; Shishehbori and Awan, 2024; Mansoori et al., 2024; van Os et al., 2023; Cai et al., 2024; Krittanawong et al., 2020; Zhao et al., 2021; Terry et al., 2024). Despite these advances, two major gaps persist. First, most CVD prediction studies employ a single feature-selection strategy—typically univariate ranking or tree-based importance—without comparing distinct paradigms, even though feature selection strongly influences both model performance and interpretability. Recent research underscores the importance of evaluating stability-based methods (e.g., resampling or stability selection) (Meinshausen and Bühlmann, 2010) and structure-aware paradigms such as hypergraph-based feature selection, which can capture multi-way relationships among features (Yang and Wu, 2023; Misiorek and Janowski, 2023; Qu et al., 2024; Jin et al., 2023). Second, although many machine learning models achieve high predictive accuracy, few have been deployed as open, web-accessible, and reproducible tools that unite interpretability with clinical usability.

While personalized predictive modeling has advanced considerably, significant challenges remain in fully understanding the complex relationships among contributing factors and in tailoring the most effective treatment strategies for individual patients. A wide range of socio-demographic, behavioral, and clinical factors contribute to variability in CVD outcomes, including age, gender, race or Hispanic origin, education, socioeconomic status, smoking history, physical activity, sleep duration, diabetes status, body mass index, blood pressure, lipid and glycemic profiles, inflammatory and hematological markers, and medication use related to blood pressure and cholesterol control. The complexity and interplay of these variables highlight the need for data-driven models capable of capturing such nuances. We hypothesize that machine learning methods can uncover and rank the most influential factors in predicting CVD risk, offering an objective and individualized framework for risk evaluation. Such a model has the potential to assist healthcare professionals in selecting the most suitable, patient-specific treatment plans to improve outcomes.

Unlike prior studies that primarily emphasized performance, this work integrates a robust feature-selection paradigm with SHAP-driven interpretability into an interactive, web-based framework, addressing the gap between algorithmic explainability and clinical usability. Using data from the NHANES, we developed a machine learning pipeline for CVD risk prediction that integrates the optimal feature evaluation method with interpretable model assessment. By quantifying feature contributions, this framework helps prioritize the most influential clinical, lifestyle, and demographic factors for enhancing cardiovascular risk assessment and guiding individualized treatment planning. This approach supports evidence-based decision-making by health professionals and improves treatment outcomes for patients at risk of CVD. The publicly available web application provides predictive outputs, probability scores, and SHAP-based visualizations. It also supports batch analysis and continuous integration of new data to further enhance model performance and adaptability.

## Methods

**Figure 1:**
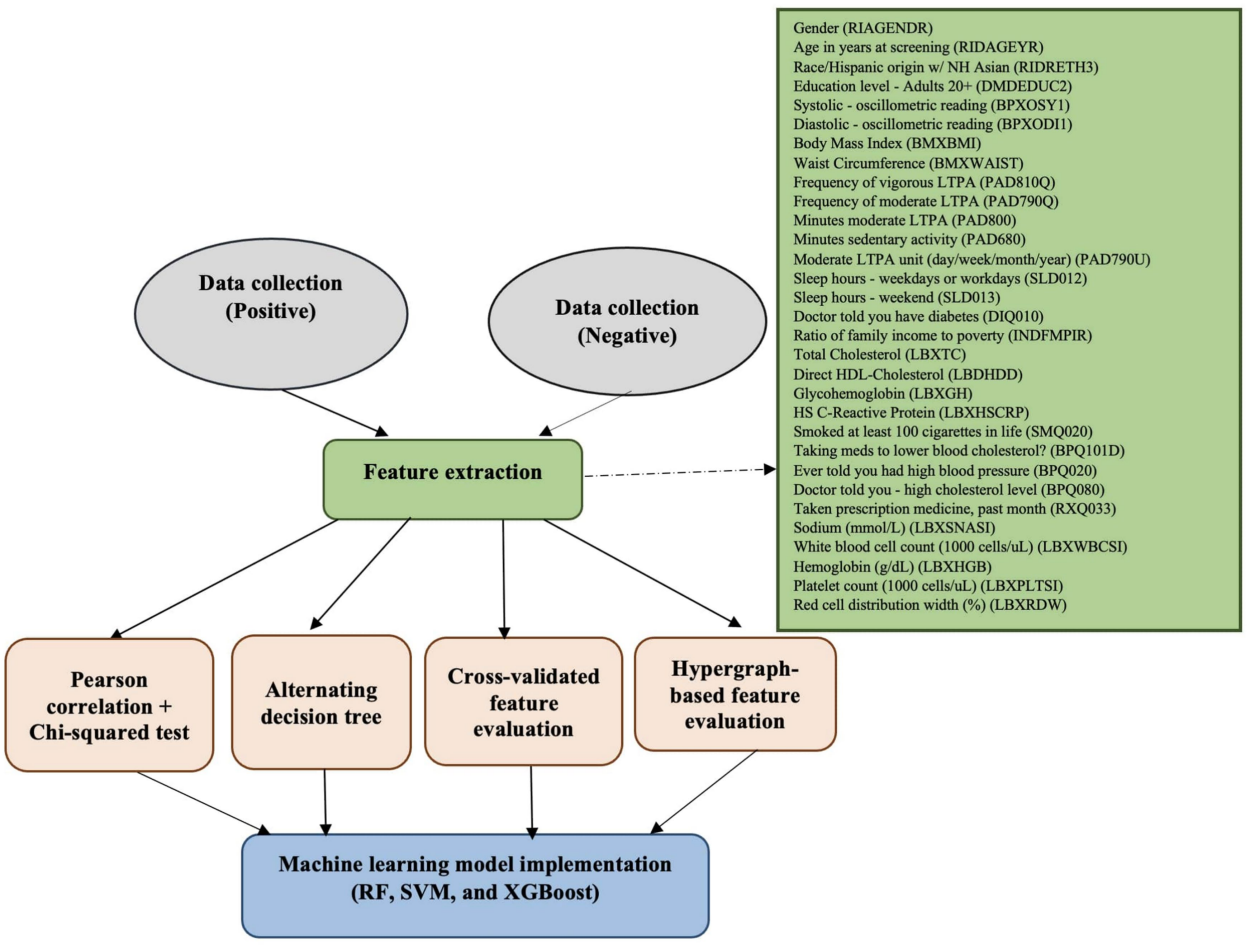
Overview of the procedure for detecting cardiovascular risk.

Figure 1 outlines the full workflow of our proposed methodology. The process begins with collecting data from individuals classified as either at risk for CVD or not at risk. From this data, a pool of candidate features is generated. We then apply multiple feature evaluation techniques—including Pearson correlation + Chi-squared test (Pearson, 1896; Pearson, 1900), Alternating Decision Tree (ADT) (Akhter and Miller, 2024; Freund and Mason, 1999), Cross-Validated Feature Evaluation (CVFE) (Yang and Wu, 2023), and Hypergraph-Based Feature Evaluation (HFE) (Misiorek and Janowski, 2023)—to eliminate features with low relevance or minimal impact. The refined feature subsets are subsequently utilized to train random forest (RF), support vector machine (SVM), and Extreme Gradient Boosting (XGBoost) models, which are evaluated for their predictive performance.

### Data and features

The data for this study were sourced from the NHANES, a publicly available national health survey (Terry et al., 2024). The analysis used demographics, examination, laboratory, and questionnaire data collected between August 2021 and August 2023 as part of the NHANES 2021–2023 cycle, which provides updated, post-pandemic information on the health and nutritional status of the U.S. civilian, noninstitutionalized population. NHANES is a cross-sectional survey, and therefore no longitudinal follow-up data are available. Each year, approximately 5,000 participants from 15 survey locations across the United States were selected through a multistage, probability-based sampling design to ensure national representativeness. Data were obtained through household interviews and standardized health examinations conducted in Mobile Examination Centers (MECs), which traveled to selected counties for roughly 9 weeks per site.

Participants aged 20 years or older with complete data on CVD outcomes and predictor variables were included in this study. Those who were institutionalized, on active military duty, or had missing responses for key demographic, laboratory, or clinical variables were excluded. For any feature with more than 30% missing data, that variable was removed from the analysis to ensure data quality and consistency. We considered only records that were complete for all retained features. The outcome variable, CVD status, was defined based on self-reported, physician-diagnosed stroke, heart attack, coronary heart disease, or heart failure. This definition of CVD status follows the CDC guidelines for NHANES analytic studies, where cardiovascular outcomes are based on self-reported, physician-diagnosed conditions. Although this approach does not involve adjudicated clinical records or imaging confirmation, it has been widely used and validated in prior epidemiological and machine-learning investigations employing NHANES and similar large-scale survey datasets (Martin-Morales et al., 2023; Dinh et al., 2019), demonstrating their utility for population-level cardiovascular-risk modeling.

The final dataset comprised 335 individuals with CVD and 3,187 individuals without CVD. To mitigate class imbalance, a random undersampling approach was applied to the non-CVD group, resulting in a balanced dataset of 335 CVD and 335 non-CVD participants. This balancing was conducted exclusively for predictive modeling to ensure fair classifier evaluation and stable performance metrics. The balanced dataset does not reflect population-level CVD prevalence and was not used for causal inference. Therefore, all performance metrics and feature contributions represent predictive associations within the analytic dataset rather than causal effects.

The dataset was randomly divided into 80% training and 20% testing subsets using stratified sampling to maintain proportional representation of CVD and non-CVD participants. This approach was consistently applied across all feature selection strategies to ensure a fair comparison of model performance under identical data partitions. All preprocessing and feature-selection steps were conducted exclusively on the training subset to prevent data leakage, and model evaluation was performed on the independent test subset. A total of 31 features were initially considered as candidate predictors and are listed in Supplementary Table S1. Table 1 summarizes the demographic and clinical features, their categorical groupings, distributions, and corresponding *p*-values, with *p* < 0.05 denoting statistical significance.

**Table 1:** Summary of the demographic profile and clinical features of the study population.

Characteristics		All subjects (*N* = 670)	CVD risk (*N* = 335)	No CVD risk (*N* = 335)	*p*-value
SMQ020 - Smoked at least 100 cigarettes in life, *N* (%)	1 (Yes)	338 (50.45)	213 (63.58)	125 (37.31)	<0.0001^a^
	2 (No)	332 (49.55)	122 (36.42)	210 (62.69)	
PAD810Q - Frequency of vigorous LTPA, Median (IQR)		5.39760534693403e-79 (5.39760534693403e-79, 2.0)	5.39760534693403e-79 (5.39760534693403e-79, 2.0)	1.0 (5.39760534693403e-79, 2.0)	<0.0001^b^
PAD790Q - Frequency of moderate LTPA, Median (IQR)		3 (2, 4)	3 (2, 4)	3 (2, 4)	<0.0001^b^
PAD790U - Moderate LTPA unit, *N* (%)	D (Day)	91 (13.58)	56 (16.72)	35 (10.45)	0.1054^a^
	W (Week)	516 (77.01)	246 (73.43)	270 (80.6)	
	M (Month)	52 (7.76)	27 (8.06)	25 (7.46)	
	Y (Year)	11 (1.64)	6 (1.79)	5 (1.49)	
PAD800 - Minutes moderate LTPA, Median (IQR)		45 (30, 60)	45 (30, 60)	45 (30, 60)	<0.0001^b^
PAD680 - Minutes sedentary activity, Median (IQR)		300 (240, 480)	300 (210, 480)	360 (240, 480)	<0.0001^b^
SLD012 - Sleep hours - weekdays or workdays, *N* (%)	3 to 13.5 (Range of values)	665 (99.25)	330 (98.51)	35 (100)	0.0806^a^
	2 (Less than 3 h)	3 (0.45)	3 (0.90)	0 (0.00)	
	14 (14 h or more)	2 (0.30)	2 (0.60)	0 (0.00)	
SLD013 - Sleep hours - weekends, *N* (%)	3 to 13.5 (Range of values)	666 (99.40)	333 (99.40)	333 (99.40)	1^a^
	2 (Less than 3 h)	2 (0.30)	1 (0.30)	1 (0.30)	
	14 (14 h or more)	2 (0.30)	1 (0.30)	1 (0.30)	
DIQ010 - Doctor told you have diabetes, *N* (%)	1 (Yes)	129 (19.25)	96 (28.66)	33 (9.85)	<0.001^a^
	2 (No)	511 (76.27)	220 (65.67)	291 (86.87)	
	3 (Borderline)	30 (4.48)	19 (5.67)	11 (3.28)	
RIDAGEYR - Age in years at screening, *N* (%)	0 to 79 (Range of values)	619 (92.39)	291 (86.87)	328 (97.91)	<0.001^a^
	80 (80 Years of age and over)	51 (7.61)	44 (13.13)	7 (2.09)	
RIAGENDR – Gender, *N* (%)	1 (Male)	337 (50.3)	207 (61.79)	130 (38.81)	<0.001^a^
	2 (Female)	333 (49.7)	128 (38.21)	205 (61.19)	
RIDRETH3 - Race/Hispanic origin w/ NH Asian, *N* (%)	1 (Mexican American)	28 (4.18)	8 (2.39)	20 (5.97)	0.0792^a^
	2 (Other Hispanic)	58 (8.66)	27 (8.06)	31 (9.25)	
	3 (Non-Hispanic White)	425 (63.43)	220 (65.67)	205 (61.19)	
	4 (Non-Hispanic Black)	67 (10.0)	38 (11.34)	29 (8.66)	
	6 (Non-Hispanic Asian)	38 (5.67)	14 (4.18)	24 (7.16)	
	7 (Other Race - Including Multi-Racial)	54 (8.06)	28 (8.36)	26 (7.76)	
DMDEDUC2 - Education level - Adults 20+, *N* (%)	1 (Less than 9th grade)	27 (4.03)	21 (6.27)	6 (1.79)	<0.001^a^
	2 (9-11th grade (Includes 12th grade with no diploma))	51 (7.61)	35 (10.45)	16 (4.78)	
	3 (High school graduate/GED or equivalent)	132 (19.7)	76 (22.69)	56 (16.72)	
	4 (Some college or AA degree)	209 (31.19)	111 (33.13)	98 (29.25)	
	5 (College graduate or above)	251 (37.46)	92 (27.46)	159 (47.46)	
INDFMPIR - Ratio of family income to poverty, *N* (%)	0 to 4.99 (Range of values)	488 (72.84)	266 (79.40)	222 (66.27)	0.0002^a^
	5 (Value greater than or equal to 5.00)	182 (27.16)	69 (20.60)	113 (33.73)	
BMXBMI - Body Mass Index (kg/m^2^), Median (IQR)		28.70 (25.02, 33.10)	28.90 (25.40, 33.40)	28.30 (24.50, 33.05)	<0.001^b^
BMXWAIST - Waist Circumference (cm), Median (IQR)		101.35 (91.03, 112.47)	104.00 (93.75, 115.90)	99.60 (88.30, 110.35)	<0.001^b^
BPXOSY1 - Systolic - oscillometric reading, Median (IQR)		122.00 (111.00, 135.00)	127.00 (114.00, 140.00)	117.00 (109.00, 129.00)	<0.001^b^
BPXODI1 - Diastolic - oscillometric reading, Median (IQR)		75.00 (67.00, 82.00)	74.00 (66.00, 82.00)	76.00 (68.00, 81.50)	<0.001^b^
LBXTC - Total Cholesterol (mg/dL), Median (IQR)		177.00 (146.00, 206.75)	158.00 (134.00, 189.50)	193.00 (165.50, 216.50)	<0.001^b^
LBDHDD - Direct HDL-Cholesterol (mg/dL) - Total Cholesterol (mg/dL), Median (IQR)		51.00 (43.00, 61.00)	49.00 (42.00, 59.00)	54.00 (45.00, 64.50)	<0.001^b^
LBXGH - Glycohemoglobin (%), Median (IQR)		5.60 (5.30, 6.00)	5.80 (5.50, 6.40)	5.40 (5.20, 5.80)	<0.001^b^
LBXHSCRP - HS C-Reactive Protein (mg/L), Median (IQR)		1.77 (0.85, 4.29)	1.64 (0.83, 4.30)	1.92 (0.90, 4.28)	<0.001^b^
BPQ101D - Taking meds to lower blood cholesterol? *N* (%)	1 (Yes)	311 (46.42)	242 (72.24)	69 (20.6)	<0.001^a^
	2 (No)	359 (53.58)	93 (27.76)	266 (79.4)	
BPQ020 - Ever told you had high blood pressure, *N* (%)	1 (Yes)	326 (48.66)	235 (70.15)	91 (27.16)	<0.001^a^
	2 (No)	344 (51.34)	100 (29.85)	244 (72.84)	
BPQ080 - Doctor told you - high cholesterol level, *N* (%)	1 (Yes)	380 (56.72)	250 (74.63)	130 (38.81)	<0.001^a^
	2 (No)	290 (43.28)	85 (25.37)	205 (61.19)	
RXQ033 - Taken prescription medicine, past month, *N* (%)	1 (Yes)	551 (82.24)	324 (96.72)	227 (67.76)	<0.001^a^
	2 (No)	119 (17.76)	11 (3.28)	108 (32.24)	
LBXSNASI - Sodium (mmol/L), Median (IQR)		139.00 (138.00, 141.00)	139.00 (138.00, 141.00)	139.00 (138.00, 141.00)	<0.001^b^
LBXWBCSI - White blood cell count (1,000 cells/uL), Median (IQR)		6.60 (5.70, 7.88)	6.70 (5.70, 7.90)	6.60 (5.70, 7.80)	<0.001^b^
LBXHGB - Hemoglobin (g/dL), Median (IQR)		14.00 (13.00, 14.88)	13.90 (13.00, 14.80)	14.00 (12.95, 14.90)	<0.001^b^
LBXPLTSI - Platelet count (1,000 cells/uL), Median (IQR)		239.00 (199.00, 283.00)	230.00 (185.00, 273.00)	250.00 (212.50, 293.50)	<0.001^b^
LBXRDW - Red cell distribution width (%), Median (IQR)		13.60 (13.10, 14.30)	13.80 (13.30, 14.60)	13.50 (13.00, 14.00)	<0.001^b^

a Chi-squared test.

b Wilcoxon two-sample test.

*N*, Number of individuals; LTPA, leisure-time physical activity, IQR, interquartile range, 25 and 75%.

### Assessment of predictive features

To build an effective predictive model, it is vital to discard non-contributory features during the preprocessing stage. In our study, we independently applied four feature reduction techniques—Pearson correlation + Chi-squared test (Pearson, 1896; Pearson, 1900), ADT (Akhter and Miller, 2024; Freund and Mason, 1999), CVFE (Yang and Wu, 2023), and HFE (Misiorek and Janowski, 2023)—to refine the original feature set by eliminating attributes with limited predictive value.

In the Pearson correlation + Chi-squared test approach, redundant features were removed through a two-step filtering process. First, Pearson’s correlation coefficient was calculated among continuous variables to identify highly correlated pairs; when the absolute correlation coefficient was ≥ 0.90, one of the correlated features was removed. Second, the Chi-squared test was applied to categorical variables to assess pairwise associations, and for feature pairs with *p* ≤ 0.001, one variable was retained while the other was excluded. This traditional filter approach served as a reference to benchmark the performance and stability of the more advanced feature-evaluation strategies. A total of 26 out of 31 features were ultimately retained through this selection procedure, as detailed in Supplementary Table S2.

The ADT approach merges the straightforward, interpretable form of a conventional decision tree with the performance enhancements derived from boosting algorithms. This technique structures its model using decision tree stumps, which are foundational units commonly associated with boosting. One of the notable advantages of ADT is its flexible branching structure; unlike traditional trees with mutually exclusive paths, ADT allows overlapping routes, enabling multiple decision paths to contribute simultaneously to a prediction. The structure begins with a prediction node that assigns a numerical score, followed by layers of decision nodes that contain conditions used to evaluate input features. These layers alternate in a pattern—prediction nodes followed by decision nodes and vice versa. Decision nodes apply specific logical criteria, while prediction nodes assign fixed numeric contributions to the outcome. Importantly, prediction nodes appear at both the starting point (root) and terminal ends (leaves) of the tree, underscoring the distinctive, layered composition and operational logic that sets ADT apart from conventional decision tree models.

The ADT constructs a series of classification rules, each composed of three main elements: a prerequisite condition, a logical condition, and a pair of numerical scores. The logical condition takes the form of a predicate expressed as “feature <operator> threshold,” while the prerequisite is a compound logical statement formed by combining multiple such conditions using conjunctions. These rules are evaluated hierarchically using nested “if” statements, and their respective scores are used to compute the final prediction for a data sample. The procedure initiates with a root rule defined by unconditional logic—both the prerequisite and condition are set to “true”—and corresponding scores are computed using the weights assigned to training instances. Initially, each training sample is assigned an equal weight of $\frac{1}{t}$ , where *t* denotes the total number of training examples. During the training process, the ADT algorithm repeatedly generates new rules by identifying the most effective pair of prerequisite and condition that minimizes a specific objective function, denoted as *z*. This function evaluates the discriminatory power of a rule based on its ability to separate positive and negative classes effectively. For every new rule created, updated scores are computed through a boosting-based mechanism. Training sample weights are also revised in accordance with the rule’s classification accuracy on each example, emphasizing incorrectly predicted instances to refine future splits. This iterative process continues until a predetermined stopping criterion is satisfied—such as reaching the maximum number of iterations or when further performance gains become negligible. The resulting rule set defines an alternating decision tree, where each prediction node holds a scalar value, and the tree’s topology is dictated by the prerequisite logic embedded within the constructed rules. Only a portion of the total feature set is used in the final ADT, reflecting the most relevant variables identified through this process. For implementation, we evaluated 50 randomly selected values for *B*, representing the number of boosting cycles. The final ADT model—shown in Supplementary Figure S1—demonstrates the decision structure derived from 31 candidate features. Ultimately, 15 features were retained through the ADT-based selection process, as presented in Supplementary Table S3.

**Figure 2:**
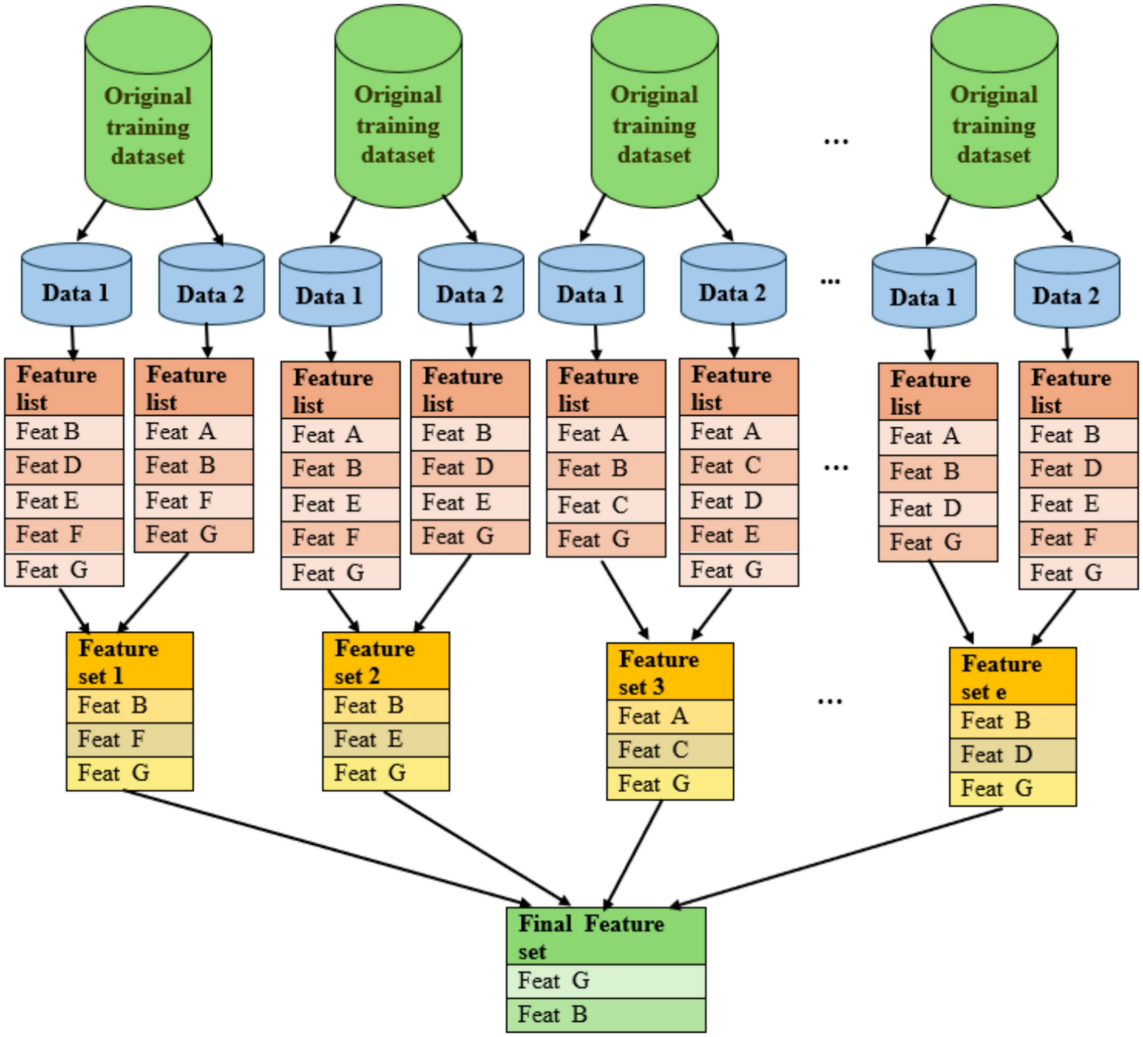
Visual representation of the feature selection process using the cross-validated feature evaluation (CVFE) method.

In addition, we incorporated both the CVFE approach and a hypergraph-based technique to refine the initial collection of features. The CVFE process is illustrated in Figure 2. Initially, the dataset was randomly divided into *c* distinct subsets. For each subset, we applied the XGBoost algorithm to determine the most influential features, optimizing model hyperparameters through a grid search procedure. Feature selection was conducted independently for every subset, followed by the construction of an intersected feature set comprising features common to all subsets. This intersection-based strategy was intentionally adopted to enhance feature stability and reproducibility rather than as an arbitrary choice. By retaining only those features that consistently appeared across multiple resampled subsets, the CVFE approach identified robust predictors less sensitive to random data partitions. This consensus-oriented process mitigates noise introduced by individual sampling variations and yields a reproducible, generalizable feature subset suitable for downstream model training. This entire process was repeated *e* times, resulting in *e* intersected feature sets. Subsequently, any feature appearing in at least (*p* × 100)% of these intersected sets was incorporated into the final selected feature list. Table 2 details the quantity of features obtained from CVFE under various parameter settings of *c*, *e*, and *p*. A comprehensive list of the selected key features can be found in Supplementary Tables S4–S7.

**Table 2:** Summary of the number of selected features and corresponding evaluation metrics—accuracy, 95% confidence intervals, precision, recall, F1 score, and AUC—on the testing dataset for different feature subsets. The most effective model for each feature set is highlighted in bold.

Feature evaluation algorithm	Configuration	Number of features	ML model	*Test_Acc_*	95% confidence interval	*Test_Precision_*	*Test_Recall_*	*Test_F1_*	*Test_AUC_*
Pearson correlation + Chi-squared test	–	26	XGBoost	0.7537	(0.6719, 0.824)	0.7361	0.7910	0.7626	0.8485
			RF	0.7537	(0.6719, 0.824)	0.7297	0.8060	07660	0.8552
			**SVM**	**0.7612**	**(0.6799, 0.8306)**	**0.7333**	**0.8209**	**0.7746**	**0.8258**
ADT	***B* = 50**	15	**XGBoost**	**0.7836**	**(0.7042, 0.85)**	**0.7568**	**0.8358**	**0.7943**	**0.8732**
			RF	0.7761	(0.6961, 0.8436)	0.7467	0.8358	0.7887	0.8793
			SVM	0.7687	(0.688, 0.8371)	0.7368	0.8358	0.7832	0.8641
CVFE	(*c* = 2, *e* = 10, *p* = 0.2)	31	XGBoost	0.791	(0.7124, 0.8564)	0.7671	0.8358	0.8000	0.8813
			**RF**	**0.8134**	**(0.737, 0.8755)**	**0.7692**	**0.8955**	**0.8276**	**0.8875**
			SVM	0.7761	(0.6961, 0.8436)	0.7403	0.8507	0.7917	0.8599
	(*c* = 2, *e* = 10, *p* = 0.6)	31	XGBoost	0.791	(0.7124, 0.8564)	0.7671	0.8358	0.8000	0.8813
			**RF**	**0.8134**	**(0.737, 0.8755)**	**0.7692**	**0.8955**	**0.8275**	**0.8875**
			SVM	0.7761	(0.6961, 0.8436)	0.7403	0.8507	0.7917	0.8599
	(*c* = 2, *e* = 5, *p* = 0.8)	28	XGBoost	0.791	(0.7124, 0.8564)	0.7532	0.8657	0.8056	0.8781
			**RF**	**0.806**	**(0.7288, 0.8692)**	**0.7662**	**0.8806**	**0.8194**	**0.8795**
			SVM	0.7687	(0.688, 0.8371)	0.7368	0.8358	0.7832	0.8594
	(*c* = 3, *e* = 5, *p* = 0.6)	28	XGBoost	0.7761	(0.6961, 0.8436)	0.7467	0.8358	0.7887	0.8819
			**RF**	**0.7985**	**(0.7205, 0.8628)**	**0.7564**	**0.8806**	**0.8138**	**0.8866**
			SVM	0.7612	(0.6799, 0.8306)	0.7333	0.8209	0.7746	0.8568
**HFE bin = 5**	*β* = 25	8	XGBoost	0.7836	(0.7042, 0.85)	0.7500	0.8507	0.7972	0.8630
			RF	0.7761	(0.6961, 0.8436)	0.7342	0.8657	0.7945	0.8646
			**SVM**	**0.806**	**(0.7288, 0.8692)**	**0.7662**	**0.8806**	**0.8194**	**0.8846**
	*β* = 50	**16**	XGBoost	0.7985	(0.7205, 0.8628)	0.7778	0.8358	0.8058	0.8915
			RF	0.806	(0.7288, 0.8692)	0.7662	0.8806	0.8194	0.9018
			**SVM**	**0.8284**	**(0.7537, 0.888)**	**0.7895**	**0.8955**	**0.8392**	**0.9027**
	*β* = 75	23	XGBoost	0.7761	(0.6961, 0.8436)	0.7403	0.7206	0.7917	0.8906
			**RF**	**0.8134**	**(0.737, 0.8755)**	**0.7692**	**0.8955**	**0.8276**	**0.8995**
			SVM	0.7836	(0.7042, 0.85)	0.7436	0.8657	0.8000	0.8866
HFE bin = 10	*β* = 25	8	XGBoost	0.7537	(0.6719, 0.824)	0.7179	0.8358	0.7724	0.8717
			RF	0.7537	(0.6719, 0.824)	0.7125	0.8507	0.7755	0.8603
			**SVM**	**0.7985**	**(0.7205, 0.8628)**	**0.7632**	**0.8657**	**0.8112**	**0.8868**
	*β* = 50	16	XGBoost	0.7836	(0.7042, 0.85)	0.7568	0.8358	0.7943	0.8882
			RF	0.806	(0.7288, 0.8692)	0.7662	0.8806	0.8194	0.8946
			**SVM**	**0.806**	**(0.7288, 0.8692)**	**0.7808**	**0.8507**	**0.8143**	**0.9076**
	*β* = 75	23	XGBoost	0.7985	(0.7205, 0.8628)	0.7703	0.8507	0.8085	0.8991
			**RF**	**0.806**	**(0.7288, 0.8692)**	**0.7595**	**0.8955**	**0.8219**	**0.8989**
			SVM	0.7836	(0.7042, 0.85)	0.7436	0.8657	0.8000	0.8866

$\mathrm{Tes}t_{\mathrm{acc}}$ : Accuracy on the testing dataset.

$\mathrm{Tes}t_{\text{precision}}$ : Precision on the testing dataset.

$\mathrm{Tes}t_{\text{recall}}$ : Recall on the testing dataset.

$\mathrm{Tes}t_{F1}$ : F1 score on the testing dataset.

$\mathrm{Tes}t_{\mathrm{AUC}}$ : AUC on the testing dataset.

*B*: Number of boosting cycles.

*c*: Number of disjoint sub-parts.

*e*: Number of repeated runs.

*p*: Proportions of repeated runs for extracting common features.

*β*: percentage of features selected.

**Figure 3:**
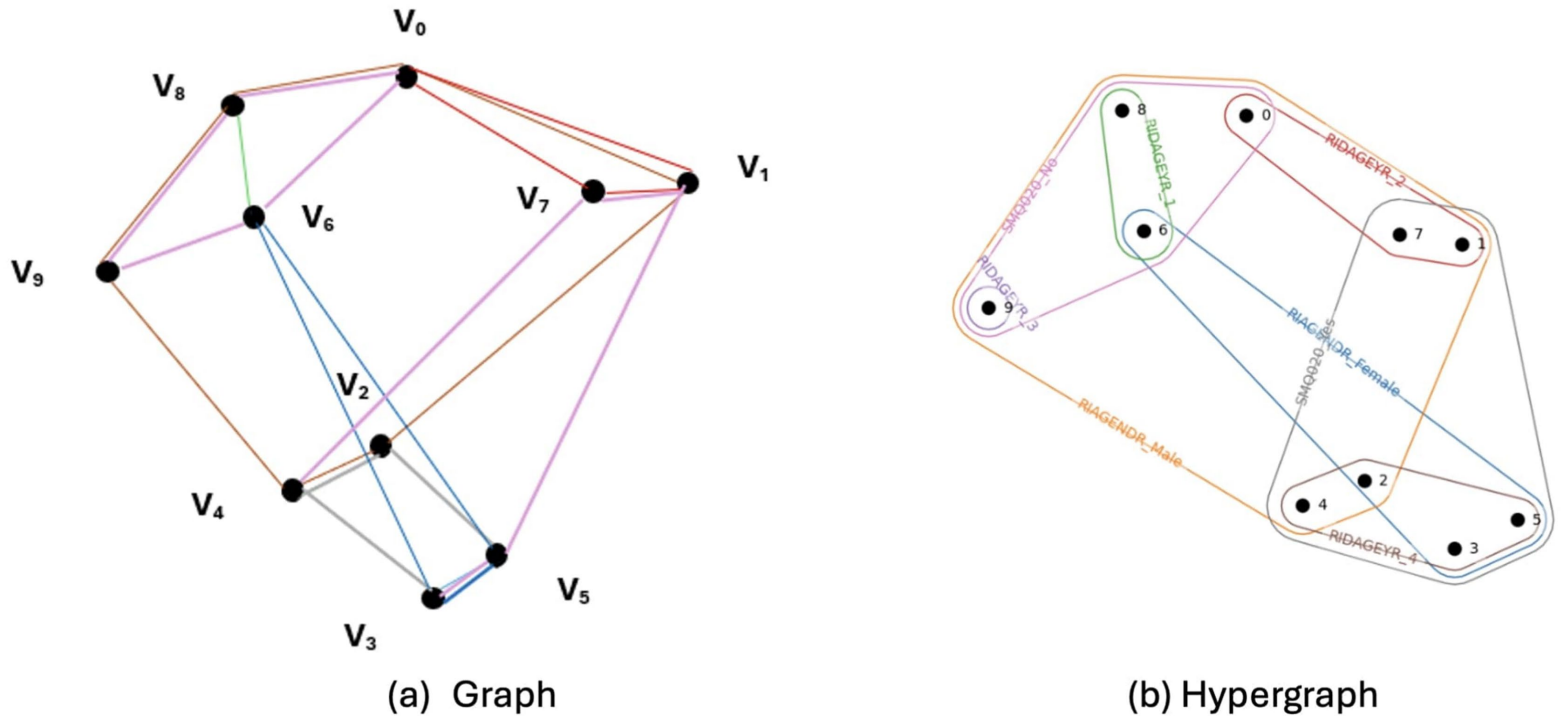
Comparison between a standard graph and a hypergraph. **(a)** A standard graph where each edge connects two vertices. **(b)** A hypergraph where each hyperedge can connect multiple vertices.

The HFE technique involves modeling feature interactions through a hypergraph structure. Unlike traditional graphs, represented as G(*V*, *E*), where vertices (*V*) are connected by pairwise edges (*E*), a hypergraph generalizes this concept by permitting each edge, termed a hyperedge, to link multiple vertices simultaneously. Formally, a hypergraph is defined as G(*V*, *E*), with *V* as the set of nodes and *E* comprising hyperedges, each of which is a subset of *V*. Figure 3 contrasts a standard graph with a hypergraph. Building on the hypergraph-based importance assessment framework proposed by Misiorek and Janowski (2023), continuous features were discretized as a preprocessing step to enable their representation within a hypergraph model originally defined for categorical feature values. Each discretized feature value was modeled as a hyperedge connecting all samples sharing that value, while class labels were represented as a partition over the vertex set. Feature-value importance was derived from random walks on the hypergraph and their contribution to hypergraph cut conductance, which quantifies how strongly a given feature value connects samples across class-label partitions. Feature-level importance scores were obtained by aggregating importance ratings across all values of a given feature, consistent with the feature aggregation strategy described in the original framework. Feature ranking was guided by the hypergraph cut conductance minimization principle, which penalizes feature values whose class distributions are proportional to overall class sizes and promotes values whose distributions deviate from class proportions. To determine the final feature subset, features were ranked according to their aggregated importance scores, and the top 𝑧 features were retained, where *z* = *β* × *m*, *m* denotes the total number of features, and 𝛽 controls the proportion of selected predictors. In our experiments, discretization settings of 5 and 10 bins were used to examine the impact of coarse versus fine value partitioning, while different values of 𝛽 were evaluated to analyze the trade-off between feature sparsity and information retention. The resulting feature counts for different 𝛽 values and bin sizes are reported in Table 2, and the specific features retained under each discretization configuration are detailed in Supplementary Tables S8, S9.

Each of the feature-selection methods employed in this study contributes differently to managing feature correlation and redundancy. The Pearson correlation filter directly identifies linear associations among continuous variables, while the Chi-squared test evaluates dependency between categorical variables and the outcome. The ADT method inherently reduces redundancy because tree-based algorithms prioritize features that provide unique information gain, thereby minimizing overlap among correlated predictors. The CVFE technique emphasizes feature stability across folds, indirectly controlling multicollinearity by retaining only features that consistently contribute to predictive performance. The HFE further extends this concept by modeling higher-order, non-linear dependencies among features, enabling detection of interactions beyond pairwise relationships. Together, these complementary approaches provide a robust framework for selecting the most informative and non-redundant predictors of CVD risk.

### Web application

**Figure 4:**
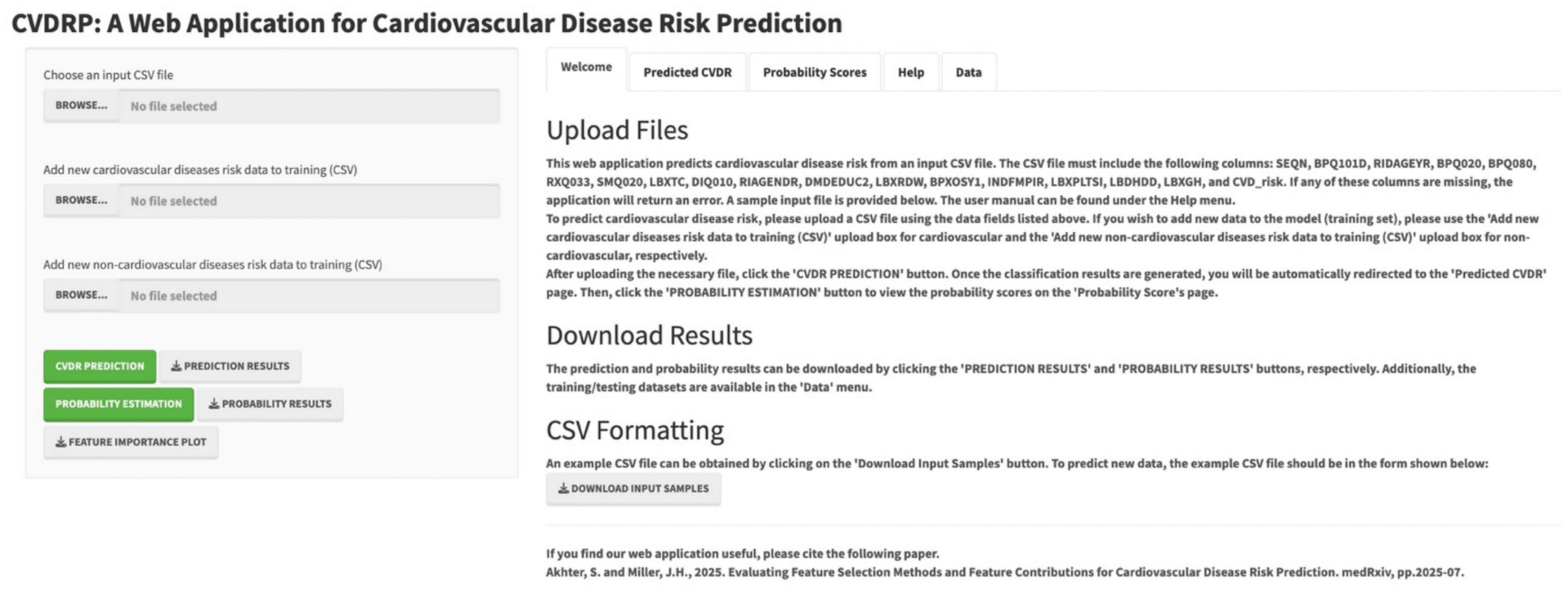
The web application for CVD risk prediction.

As shown in Figure 4, the best feature evaluation approach (HFE) was incorporated into a machine learning-powered web application. This tool delivers both classification outcomes and associated probability scores. Instructions for uploading datasets, performing binary classification, and estimating probabilities are provided within the application interface. Users have the option to download relevant output files and contribute additional training data, thereby improving model performance. The tool also supports the export of a SHAP visualization, allowing users to examine how top-ranked features influence predictions.

### Code and data availability

The scripts used in this study are available at https://github.com/suraiya14/CVDRP.

## Results

Following the reduction of the original feature set using four independent feature-selection strategies, we developed individual predictive models based on the selected features using the RF, SVM, and XGBoost algorithms. Each classifier was optimized using a grid-search procedure with 10-fold cross-validation on the training data to ensure robust hyperparameter tuning and reduce performance variance. To interpret and analyze the contribution of each feature in the best-performing model, we applied the SHAP method (Lundberg et al., 2020). SHAP is a model-independent method based on cooperative game theory that explains predictions by estimating the average marginal effect of each feature across different feature combinations. For the top-performing model, SHAP values were computed using a kernel-based SHAP approach, which provides an approximation of Shapley values for nonlinear classifiers that do not natively expose feature attribution scores.

### Performance assessment

The predictive models were constructed by utilizing different sets of features selected through Pearson correlation + Chi-squared test, ADT, CVFE, and HFE methodologies in conjunction with the training data. The model’s performance was assessed on the test data using the metrics defined in Equations 1–4, where TP, TN, FP, and FN represent true positive, true negative, false positive, and false negative counts, respectively. Among these evaluation metrics, accuracy was employed to determine the ratio of correctly predicted outcomes relative to the overall number of samples in the dataset.


$$\mathrm{Tes}t_{\mathrm{Acc}}=\frac{\mathrm{TP}+\mathrm{TN}}{\mathrm{TP}+\mathrm{TN}+\mathrm{FP}+\mathrm{FN}}$$ (1)

$$\mathrm{Tes}t_{\text{recall}}=\frac{\mathrm{TP}}{\mathrm{TP}+\mathrm{FN}}$$ (2)

(3) $$\mathrm{Tes}t_{\text{precision}}=\frac{\mathrm{TP}}{\mathrm{TP}+\mathrm{FP}}$$

(4) $$\mathrm{Tes}t_{F1}=2\times \frac{\left(\mathrm{Tes}t_{\text{precision}}\times \mathrm{Tes}t_{\text{recall}}\right)}{\left(\mathrm{Tes}t_{\text{precision}}+\mathrm{Tes}t_{\text{recall}}\right)}$$

In addition, we computed recall and precision to further evaluate model performance. Recall quantifies the ability of the model to correctly detect actual positive cases, whereas precision reflects the proportion of positive predictions that are genuinely correct. To provide a balanced evaluation that incorporates both precision and recall, we utilized the F1 score, which is defined as their harmonic mean. Moreover, the Area Under the Curve (AUC) metric was used to assess the effectiveness of the binary classifier. Higher AUC values correspond to stronger model performance, with a score of 1 indicating flawless classification and 0.5 representing performance equivalent to random guessing. We also computed 95% confidence intervals for the model outputs to represent the range within which the true performance measure is expected to fall. Wider intervals correspond to greater uncertainty in the model’s predictive estimates.

Table 2 presents a detailed comparison of model performance using feature subsets selected through the Pearson correlation + Chi-squared test, ADT, CVFE, and HFE techniques. Corresponding confusion matrices for each reduced feature set are visualized in Supplementary Figure S2. The machine learning models employing features selected through the Pearson correlation + Chi-squared filter achieved moderate predictive performance. In contrast, the ADT, CVFE, and HFE methods provided enhanced predictive performance. Overall, the HFE feature set (bin = 5, *β* = 50) combined with SVM model yielded superior predictive performance compared to the ADT and CVFE sets, with the best model successfully identifying 60 out of 67 patients with CVD.

### Features identified using the HFE approach

**Figure 5:**
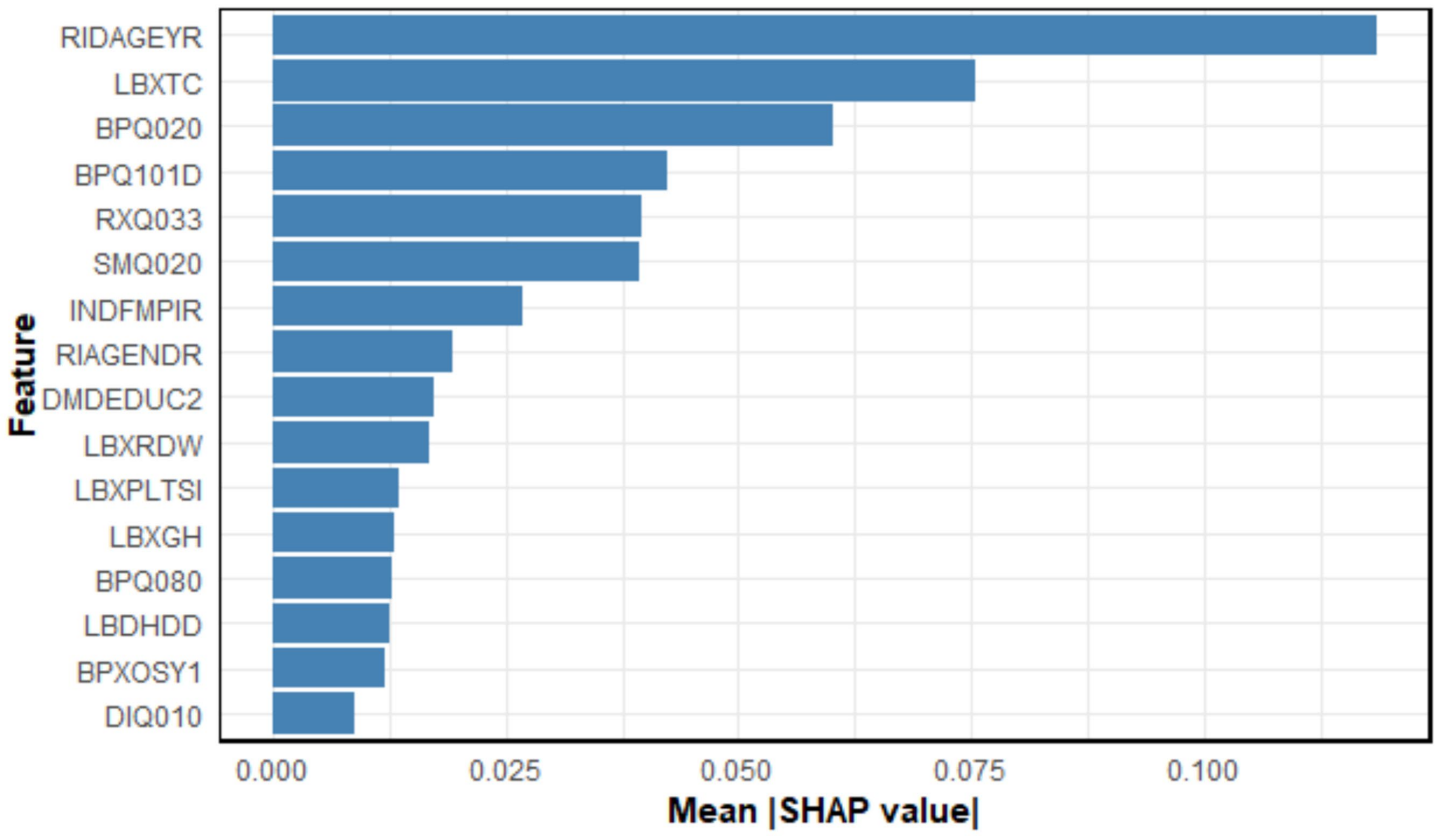
Importance of features selected by the SVM model shown as a bar chart. The *x*-axis represents the mean absolute SHAP value for each feature, reflecting its average contribution to the model’s predictions. Features are arranged from most to least influential.

As mentioned earlier, the highest predictive performance was attained using the SVM algorithm paired with the HFE feature selection method configured with bin = 5 and *β* = 50. Figure 5 shows a SHAP summary bar chart illustrating the ranking of selected features according to their importance derived from SHAP value analysis for this model. The top 10 most influential features ranked by their SHAP values were RIDAGEYR (age in years at screening), LBXTC (total cholesterol, mg/dL), BPQ020 (ever told you had high blood pressure), BPQ101D (taking medication to lower blood cholesterol), RXQ033 (taken prescription medicine in the past month), SMQ020 (smoked at least 100 cigarettes in life), INDFMPIR (ratio of family income to poverty), RIAGENDR (gender), DMDEDUC2 (education level, adults aged 20 and above), and LBXRDW (red cell distribution width, %).

It is important to note that the SHAP analysis was employed solely to improve model interpretability by quantifying each feature’s relative contribution to the prediction outcomes. The SHAP values capture associational relationships between features and predicted CVD risk rather than causal effects. Because the NHANES dataset is cross-sectional and observational, causal inference cannot be drawn from these results. The SHAP findings are therefore intended to provide interpretive insight into the model’s behavior and to guide hypothesis generation for future longitudinal or causal investigations.

### Feature impact assessment

**Figure 6:**
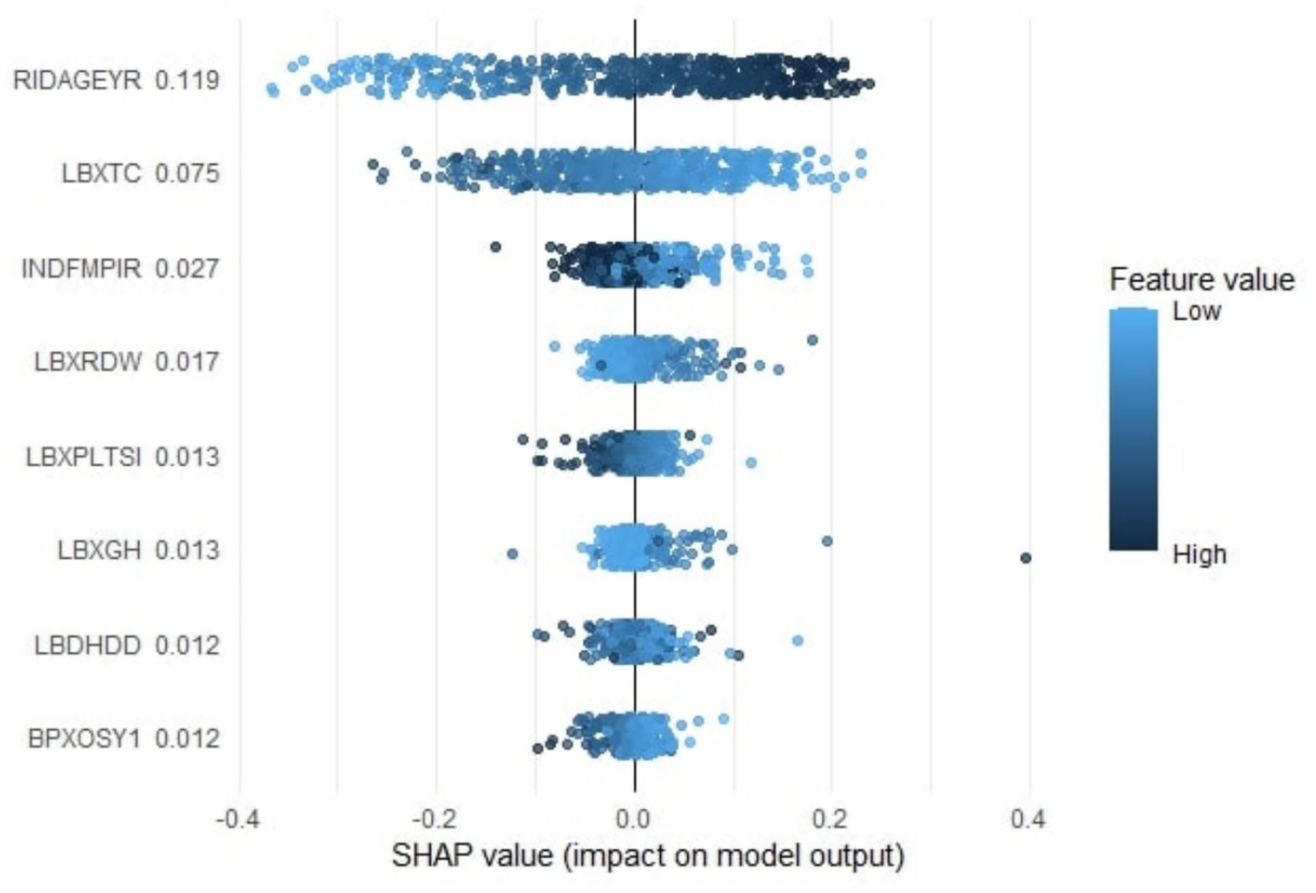
SHAP analysis illustrating the contribution and direction of the continuous features to model predictions.

Figure 6 illustrates the contributions and directions of influence of the continuous predictors identified in Figure 5, based on SHAP value analysis. Each point represents an individual participant, with overlapping points jittered to show data density. The *x*-axis indicates SHAP values, where positive values (right) increase the model predicted likelihood of CVD and negative values (left) decrease it. The color gradient represents feature magnitude, with dark blue indicating high feature values and light blue indicating low feature values.

RIDAGEYR (age) shows that higher age values correspond to positive SHAP values, indicating that older individuals have a higher predicted CVD risk, which agrees with established epidemiologic evidence. LBXTC (total cholesterol) displays an inverse pattern, where higher cholesterol values correspond to negative SHAP values and lower predicted CVD risk. This unexpected direction, often referred to as the cholesterol paradox, has been observed in older or treated populations and may reflect survivor bias or treatment effects rather than a true protective influence (Orkaby, 2020; Majnarić et al., 2021). INDFMPIR (family income to poverty ratio) indicates that higher income ratios correspond to negative SHAP values and lower predicted CVD risk, whereas lower ratios are associated with positive SHAP values and higher predicted risk, consistent with the adverse cardiovascular impact of socioeconomic disadvantage. LBXRDW (red cell distribution width) shows that higher values are associated with positive SHAP values, indicating that greater red cell heterogeneity increases predicted CVD risk. This finding aligns with epidemiologic evidence demonstrating that elevated RDW is independently associated with cardiovascular morbidity and mortality in diverse populations (Danese et al., 2015; Patel et al., 2009; Shantsila et al., 2023). LBXPLTSI (platelet count) shows that higher platelet values correspond to negative SHAP values and lower predicted CVD risk, while lower platelet values correspond to positive SHAP values and higher predicted risk. This inverse pattern may reflect platelet consumption or systemic inflammation rather than a true protective effect of reduced platelet levels. Similar non-linear or inverse associations have been reported in population studies, where low platelet counts were linked with greater cardiovascular morbidity and mortality, likely reflecting inflammation and disease burden rather than protection (Vinholt et al., 2016; Fawzy et al., 2019). LBXGH (glycohemoglobin) displays higher values aligned with positive SHAP values, confirming that elevated HbA1c levels increase the predicted CVD probability. LBDHDD (high-density lipoprotein cholesterol) generally shows that lower HDL values correspond to positive SHAP values and higher predicted cardiovascular disease risk, whereas higher HDL values are mostly associated with negative SHAP values and lower risk. However, a subset of both low and high HDL observations exhibited opposite SHAP directions, indicating that the relationship between HDL and predicted CVD is non-linear and context-dependent. Such bidirectional effects likely reflect complex interactions between HDL and other metabolic or hemodynamic factors considered by the model. BPXOSY1 (systolic blood pressure) shows that higher systolic pressure values correspond to negative SHAP values, suggesting a lower predicted CVD probability, whereas lower pressures are linked with positive SHAP values. This inverse pattern does not imply a protective effect of elevated blood pressure but may instead reflect the J-shaped association observed in treated or frail older adults, where excessively low systolic pressure relates to increased cardiovascular events and mortality due to antihypertensive overtreatment or comorbid burden (Shantsila et al., 2023; Rodriguez et al., 2014).

**Figure 7:**
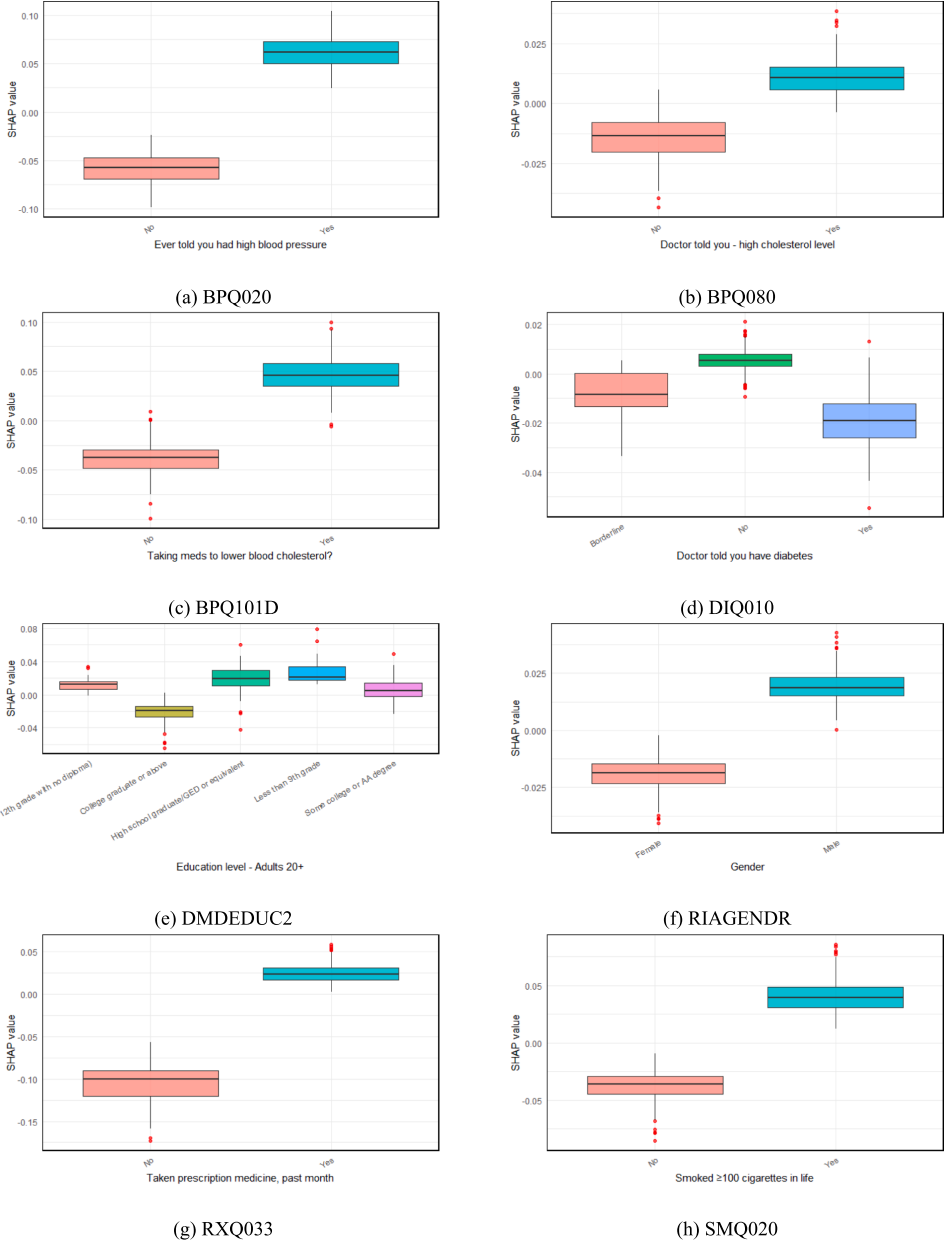
SHAP analysis of categorical predictors influencing CVD risk. **(a)** BPQ020 (ever told had high blood pressure), **(b)** BPQ080 (doctor told—high cholesterol level), **(c)** BPQ101D (taking medication to lower blood cholesterol), **(d)** DIQ010 (doctor told you have diabetes), **(e)** DMDEDUC2 (education level for adults aged 20+), **(f)** RIAGENDR (gender), **(g)** RXQ033 (taken prescription medicine in the past month), and **(h)** SMQ020 (smoked at least 100 cigarettes in life). Each subfigure shows the distribution of SHAP values for the corresponding categorical feature, indicating its contribution to the model’s prediction of CVD risk.

Figure 7 illustrates the SHAP-based evaluation of key categorical predictors influencing CVD risk, with each subfigure presenting boxplots of SHAP values for distinct categorical features, where higher SHAP values represent stronger positive contributions to the predicted likelihood of CVD and vice versa. As shown in Figures 7a–c, participants who were ever told they had high blood pressure (BPQ020 = Yes), were informed of having high cholesterol (BPQ080 = Yes), or were taking cholesterol-lowering medication (BPQ101D = Yes) exhibited notably higher SHAP values, indicating greater predicted CVD risk. Figure 7d shows that SHAP values for individuals with diabetes (DIQ010 = Yes) were broadly distributed and did not consistently exceed those of non-diabetic participants. This indicates that diabetes status, although a well-established risk factor for cardiovascular disease, exerted only a modest independent influence in the current model. Such attenuation may reflect collinearity with other metabolic predictors—particularly glycohemoglobin (LBXGH) and lipid parameters—or the influence of effective glycemic control and treatment among participants. This observation is consistent with previous studies suggesting that diabetes-related cardiovascular risk varies substantially depending on comorbidity burden and glycemic management (Rawshani et al., 2018; Einarson et al., 2018). As shown in Figure 7e, higher education levels (DMDEDUC2) were associated with lower SHAP values, suggesting an inverse relationship between educational attainment and CVD risk. Figure 7f indicates modest sex-based differences (RIAGENDR), with males showing slightly higher predicted risk overall. In Figure 7g, individuals who reported taking prescription medications within the past month (RXQ033 = Yes) had higher SHAP values, which may reflect the presence of underlying health conditions associated with increased CVD risk. Finally, Figure 7h shows that participants who had smoked at least 100 cigarettes in their lifetime (SMQ020 = Yes) had elevated SHAP values, reinforcing smoking’s well-established association with cardiovascular risk.

## Discussion

Accurate individualized prediction of CVD risk is essential for prevention, early intervention, and improved clinical outcomes. In this study, we developed an interpretable, web-accessible machine learning framework that combines HFE with SHAP to prioritize predictive features and explain model behavior. Among all evaluated combinations of feature-selection strategy and classifier, the SVM trained on the HFE-selected features (bin = 10, *β* = 50) achieved the best overall performance on the held-out test set. This demonstrates the advantage of HFE in capturing complex feature dependencies that conventional pairwise methods overlook. Coupled with SHAP-based interpretability, the framework delivers both strong predictive accuracy and transparent, clinically meaningful explanations.

These findings align with recent studies demonstrating that nonlinear machine learning algorithms, including support vector machines, random forests, and XGBoost, achieve superior predictive accuracy for cardiovascular and coronary artery disease risk compared with conventional statistical or regression-based scores (Azmi et al., 2022; DeGroat et al., 2024; Peng et al., 2023; Cai et al., 2024; Krittanawong et al., 2020; Rahim et al., 2021; Wang J. et al., 2024). Unlike traditional regression approaches that rely on prespecified functional forms, these algorithms flexibly capture complex, higher-order interactions among demographic, clinical, and biochemical variables. At the same time, growing evidence emphasizes that model interpretability is essential for clinical acceptance. SHAP explanation frameworks, in particular, have proven valuable for quantifying feature contributions and illustrating how physiological and behavioral factors influence cardiovascular risk predictions (Peng et al., 2023; Lundberg et al., 2020; Wang J. et al., 2024).

Our SHAP analysis highlighted the dominant role of metabolic, hemodynamic, and behavioral domains in driving cardiovascular risk, emphasizing glycemic control, lipid metabolism, blood pressure regulation, and smoking behavior rather than isolated biomarkers. These findings are consistent with large-scale epidemiologic and mechanistic studies linking metabolic dysregulation, hypertension, and tobacco exposure to elevated CVD risk (Singh et al., 2013; Consortium GCR, 2023; Rawshani et al., 2019; Joseph et al., 2017). Importantly, socioeconomic and behavioral indicators, including education level and family income to poverty ratio, also emerged as influential features, echoing prior work showing that social determinants and lifestyle factors substantially modulate cardiometabolic outcomes in U.S. populations (Zhao et al., 2021; Zhou et al., 2024). Together, these results underscore the value of population-based resources such as NHANES, which integrate demographic, laboratory, behavioral, and socioeconomic dimensions to support interpretable, data-driven cardiovascular-risk modeling.

Methodologically, this work contributes by operationalizing hypergraph-based feature evaluation within an explainable CVD-risk-prediction pipeline. Hypergraph frameworks extend conventional feature modeling by allowing each “edge” to connect multiple variables simultaneously, thereby capturing higher-order relationships beyond pairwise correlations (Misiorek and Janowski, 2023; Jin et al., 2023). Prior studies in biomedical prediction have demonstrated that such hypergraph learning effectively represents complex structures in heterogeneous data (Jin et al., 2023). By integrating HFE with SHAP, the proposed framework maintains interpretability by providing explicit attributions for individual features while leveraging these richer relational patterns. This integration addresses a central challenge in clinical AI by balancing statistical rigor with practical interpretability for clinicians (Lundberg et al., 2020; Wang N. et al., 2024). Moreover, the accompanying web application delivers classification results, probability estimates, and SHAP visualizations. This design enhances reproducibility and facilitates external validation, both essential for trustworthy AI in cardiovascular and preventive medicine (DeGroat et al., 2024).

Several limitations should be noted. Although SHAP analysis enhances interpretability and model transparency, it does not establish causality between predictors and cardiovascular outcomes. The NHANES dataset is cross-sectional, and the balanced endpoint used for training was achieved through random undersampling of the majority non-CVD class and was designed for predictive fairness rather than causal estimation; thus, the relationships identified reflect statistical associations rather than mechanistic effects. Although random undersampling was used to mitigate class imbalance, alternative strategies such as class weighting or synthetic sampling methods may preserve additional information from the majority class and will be explored in future work. Model performance was evaluated using an internal stratified train–test split within a single NHANES cycle, and external validation using independent datasets or additional NHANES cycles was not performed in this study. CVD status was defined using self-reported, physician-diagnosed conditions in accordance with CDC/NHANES analytic protocols. While this approach may introduce recall bias or minor misclassification compared with adjudicated clinical records, such definitions are standard in population-based machine learning studies and have been validated in prior CVD-prediction research (Martin-Morales et al., 2023; Dinh et al., 2019). Although the feature-selection strategies collectively addressed linear and nonlinear dependencies, residual correlations may persist; future studies could incorporate dimensionality-reduction or penalization methods (e.g., PCA or elastic-net regularization) to further minimize redundancy and improve interpretability. In addition, computational performance metrics such as inference latency, throughput under concurrent user access, and detailed resource utilization were not formally benchmarked in this study. While the web design improves accessibility and encourages iterative model refinement, systematic evaluation of inference time, retraining time, scalability under concurrent access, and hardware resource requirements will be addressed in future deployment-focused work. Future work will integrate longitudinal and multi-omics data, external validation across NHANES cycles, and federated-learning approaches to improve generalizability, and will also benchmark the proposed framework against validated clinical risk models such as Framingham, SCORE, and REGICOR when applied to datasets with complete longitudinal and treatment information. Finally, although the model identifies clinically relevant predictors such as age, blood pressure, cholesterol, glycohemoglobin, diabetes, smoking, and socioeconomic indicators, it is intended to complement rather than replace established clinical risk tools. The proposed framework provides a transparent, population-level approach for prioritizing risk factors, offering outputs that remain biologically plausible and consistent with known cardiovascular physiology.

In summary, we present a transparent cardiovascular-risk-prediction framework that integrates hypergraph-based feature evaluation with SHAP explainability and deploys this pipeline in an accessible web tool. The approach demonstrated high discriminative performance on held-out test data, recovered physiologically and socially meaningful predictors, and revealed higher-order interactions among demographic, clinical, and behavioral features. These results support the potential of explainable, structure-aware machine learning to inform population-level cardiovascular-risk assessment in a reproducible and clinically interpretable manner.

## Data Availability

The datasets presented in this study can be found in online repositories. The names of the repository/repositories and accession number(s) can be found at: https://github.com/suraiya14/CVDRP.

## References

[ref1] Akhter S. Miller J. H. (2024). BPAGS: a web application for bacteriocin prediction via feature evaluation using alternating decision tree, genetic algorithm, and linear support vector classifier. Front. Bioinfor. 3:1284705. doi: 10.3389/fbinf.2023.1284705 PMC10807691 38268970

[ref2] Ambekar S Phalnikar R, editors. Disease risk prediction by using convolutional neural network. 2018 Fourth International Conference on Computing Communication Control and Automation (ICCUBEA); (2018): IEEE.

[ref3] Assmann G. Cullen P. Schulte H. (2002). Simple scoring scheme for calculating the risk of acute coronary events based on the 10-year follow-up of the prospective cardiovascular Munster (PROCAM) study. Circulation 105, 310–315. doi: 10.1161/hc0302.102575 11804985

[ref4] Azmi J. Arif M. Nafis M. T. Alam M. A. Tanweer S. Wang G. (2022). A systematic review on machine learning approaches for cardiovascular disease prediction using medical big data. Med. Eng. Phys. 105:103825. doi: 10.1016/j.medengphy.2022.103825 35781385

[ref5] Cai Y. Cai Y.-Q. Tang L.-Y. Wang Y.-H. Gong M. Jing T.-C. . (2024). Artificial intelligence in the risk prediction models of cardiovascular disease and development of an independent validation screening tool: a systematic review. BMC Med. 22:56. doi: 10.1186/s12916-024-03273-7 38317226 PMC10845808

[ref6] Consortium GCR (2023). Global effect of modifiable risk factors on cardiovascular disease and mortality. N. Engl. J. Med. 389, 1273–1285. 37632466 10.1056/NEJMoa2206916 PMC10589462

[ref7] Danese E. Lippi G. Montagnana M. (2015). Red blood cell distribution width and cardiovascular diseases. J. Thorac. Dis. 7:E402. doi: 10.3978/j.issn.2072-1439.2015.10.04 26623117 PMC4635283

[ref8] DeGroat W. Abdelhalim H. Patel K. Mendhe D. Zeeshan S. Ahmed Z. (2024). Discovering biomarkers associated and predicting cardiovascular disease with high accuracy using a novel nexus of machine learning techniques for precision medicine. Sci. Rep. 14:1. doi: 10.1038/s41598-023-50600-8 38167627 PMC10762256

[ref9] Dinh A. Miertschin S. Young A. Mohanty S. D. (2019). A data-driven approach to predicting diabetes and cardiovascular disease with machine learning. BMC Med. Inform. Decis. Mak. 19, 1–15. doi: 10.1186/s12911-019-0918-5 31694707 PMC6836338

[ref10] Einarson T. R. Acs A. Ludwig C. Panton U. H. (2018). Prevalence of cardiovascular disease in type 2 diabetes: a systematic literature review of scientific evidence from across the world in 2007–2017. Cardiovasc. Diabetol. 17:83. doi: 10.1186/s12933-018-0728-6 29884191 PMC5994068

[ref11] Elsayed HAG Syed L, editors. An automatic early risk classification of hard coronary heart diseases using Framingham scoring model. Proceedings of the Second International Conference on Internet of Things, Data and Cloud Computing (2017). doi: 10.1145/3018896.3036384

[ref12] Fawzy A. Anderson J. A. Cowans N. J. Crim C. Wise R. Yates J. C. . (2019). Association of platelet count with all-cause mortality and risk of cardiovascular and respiratory morbidity in stable COPD. Respir. Res. 20:86. doi: 10.1186/s12931-019-1059-1 31068182 PMC6507019

[ref13] Freund Y. Mason L. “The alternating decision tree learning algorithm” (PDF). Proceedings of the Sixteenth International Conference on Machine Learning (ICML ‘99). Ivan Bratko, Saso Dzeroski eds., San Francisco, CA, United States: Morgan Kaufmann Publishers Inc (1999). 124–133.

[ref14] Gil-Guillen V. Orozco-Beltran D. Maiques-Galan A. Aznar-Vicente J. Navarro J. Cea-Calvo L. . (2007). Agreement between REGICOR and SCORE scales in identifying high cardiovascular risk in the Spanish population. Rev. Esp. Cardiol. 60:1042. doi: 10.1157/13111236 17953925

[ref15] Guarneros-Nolasco L. R. Cruz-Ramos N. A. Alor-Hernández G. Rodríguez-Mazahua L. Sánchez-Cervantes J. L. (2021). Identifying the main risk factors for cardiovascular diseases prediction using machine learning algorithms. Mathematics. 9:2537. doi: 10.3390/math9202537

[ref16] Hossen M. A. Tazin T. Khan S. Alam E. Sojib H. A. Monirujjaman Khan M. . (2021). Supervised machine learning-based cardiovascular disease analysis and prediction. Math. Probl. Eng. 2021:1792201. doi: 10.1155/2021/1792201

[ref17] Jin S. Hong Y. Zeng L. Jiang Y. Lin Y. Wei L. . (2023). A general hypergraph learning algorithm for drug multi-task predictions in micro-to-macro biomedical networks. PLoS Comput. Biol. 19:e1011597. doi: 10.1371/journal.pcbi.1011597 37956212 PMC10681315

[ref18] Joseph P. Leong D. McKee M. Anand S. S. Schwalm J.-D. Teo K. . (2017). Reducing the global burden of cardiovascular disease, part 1: the epidemiology and risk factors. Circ. Res. 121, 677–694. doi: 10.1161/CIRCRESAHA.117.308903 28860318

[ref19] Khandoker A. H. Al Zaabi Y. Jelinek H. F. (2019). “What can tone and entropy tell us about risk of cardiovascular diseases?” in 2019 computing in cardiology (CinC). (Singapore: IEEE).

[ref20] Kiran P. Swathi A. Sindhu M. Manikanta Y. (2022). Effective heart disease prediction using hybrid machine learning technique. South Asian J. Eng. Technol. 12, 123–130. doi: 10.26524/sajet.2022.12.49

[ref21] Krittanawong C. Virk H. U. H. Bangalore S. Wang Z. Johnson K. W. Pinotti R. . (2020). Machine learning prediction in cardiovascular diseases: a meta-analysis. Sci. Rep. 10:16057. doi: 10.1038/s41598-020-72685-1 32994452 PMC7525515

[ref22] Lundberg S. M. Erion G. Chen H. DeGrave A. Prutkin J. M. Nair B. . (2020). From local explanations to global understanding with explainable AI for trees. Nat. Mach. Intell. 2, 56–67. doi: 10.1038/s42256-019-0138-9 32607472 PMC7326367

[ref23] Majnarić L. T. Bosnić Z. Kurevija T. Wittlinger T. (2021). Cardiovascular risk and aging: the need for a more comprehensive understanding. J Geriatric Cardiol. 18:462. doi: 10.11909/j.issn.1671-5411.2021.06.004 PMC8220387 34220975

[ref24] Mandava M. (2024). MDensNet201-IDRSRNet: efficient cardiovascular disease prediction system using hybrid deep learning. Biomed. Signal Process. Control 93:106147. doi: 10.1016/j.bspc.2024.106147

[ref25] Mansoori A. Allahyari M. Mirvahabi M. S. Tanbakuchi D. Ghoflchi S. Derakhshan-Nezhad E. . (2024). Predictive properties of novel anthropometric and biochemical indexes for prediction of cardiovascular risk. Diabetol. Metab. Syndr. 16:304. doi: 10.1016/j.bspc.2024.106147 39696688 PMC11657368

[ref26] Martin-Morales A. Yamamoto M. Inoue M. Vu T. Dawadi R. Araki M. (2023). Predicting cardiovascular disease mortality: leveraging machine learning for comprehensive assessment of health and nutrition variables. Nutrients 15:3937. doi: 10.3390/nu15183937 37764721 PMC10534618

[ref27] Meinshausen N. Bühlmann P. (2010). Stability selection. J. R. Stat. Soc. B 72, 417–473.

[ref28] Misiorek P. Janowski S. (2023). Hypergraph-based importance assessment for binary classification data. Knowl. Inf. Syst. 65, 1657–1683. doi: 10.1007/s10115-022-01786-2

[ref29] Mousa D Zayed N Yassine IA, editors. Automatic cardiac MRI localization method. 2014 Cairo International Biomedical Engineering Conference (CIBEC) (2014): IEEE.

[ref30] Neumann J. T. Thao L. T. Callander E. Chowdhury E. Williamson J. D. Nelson M. R. . (2022). Cardiovascular risk prediction in healthy older people. Geroscience 44, 403–413. doi: 10.1007/s11357-021-00486-z 34762275 PMC8810999

[ref31] Ogunpola A. Saeed F. Basurra S. Albarrak A. M. Qasem S. N. (2024). Machine learning-based predictive models for detection of cardiovascular diseases. Diagnostics 14:144. doi: 10.3390/diagnostics14020144 38248021 PMC10813849

[ref32] Orkaby A. R. (2020). The highs and lows of cholesterol: a paradox of healthy aging?. Journal of the American Geriatrics Society, 68:236–237. 31930729 10.1111/jgs.16302

[ref33] Patel K. V. Ferrucci L. Ershler W. B. Longo D. L. Guralnik J. M. (2009). Red blood cell distribution width and the risk of death in middle-aged and older adults. Arch. Intern. Med. 169, 515–523. 19273783 10.1001/archinternmed.2009.11 PMC2765040

[ref34] Patel J. TejalUpadhyay D. Patel S. (2015). Heart disease prediction using machine learning and data mining technique. Heart Dis. 7, 129–137.

[ref35] Pearson K. (1896). VII. Mathematical contributions to the theory of evolution.—III. Regression, heredity, and panmixia. Philosophical Transactions of the Royal Society of London. Series A, containing papers of a mathematical or physical character. 187, 253–318.

[ref36] Pearson K. X. (1900). On the criterion that a given system of deviations from the probable in the case of a correlated system of variables is such that it can be reasonably supposed to have arisen from random sampling. Lond. Edinb. Dubl. Phil. Mag. J. Sci. 50, 157–175. doi: 10.1080/14786440009463897

[ref37] Peng M. Hou F. Cheng Z. Shen T. Liu K. Zhao C. . (2023). Prediction of cardiovascular disease risk based on major contributing features. Sci. Rep. 13:4778. doi: 10.1038/s41598-023-31870-8 36959459 PMC10036320

[ref38] Pouriyeh S. Vahid S. Sannino G. De Pietro G. Arabnia H. Gutierrez J. (2017). “A comprehensive investigation and comparison of machine learning techniques in the domain of heart disease” in 2017 IEEE Symposium on Computers and Communications (ISCC) (IEEE).

[ref39] Pu L.-N. Zhao Z. Zhang Y.-T. (2012). Investigation on cardiovascular risk prediction using genetic information. IEEE Trans. Inf. Technol. Biomed. 16, 795–808. doi: 10.1109/titb.2012.2205009 22736654

[ref40] Qu Y. Fu K. Wang L. Zhang Y. Wu H. Liu Q. (2024). Hypergraph-based multitask feature selection with temporally constrained group sparsity learning on fMRI. Mathematics. 12:1733. doi: 10.3390/math12111733

[ref41] Rahim A. Rasheed Y. Azam F. Anwar M. W. Rahim M. A. Muzaffar A. W. (2021). An integrated machine learning framework for effective prediction of cardiovascular diseases. IEEE Access 9, 106575–106588. doi: 10.1109/ACCESS.2021.3098688

[ref42] Rawshani A. Rawshani A. Franzén S. Sattar N. Eliasson B. Svensson A.-M. . (2018). Risk factors, mortality, and cardiovascular outcomes in patients with type 2 diabetes. N. Engl. J. Med. 379, 633–644. doi: 10.1056/nejmoa1800256 30110583

[ref43] Rawshani A. Rawshani A. Sattar N. Franzén S. McGuire D. K. Eliasson B. . (2019). Relative prognostic importance and optimal levels of risk factors for mortality and cardiovascular outcomes in type 1 diabetes mellitus. Circulation 139, 1900–1912. doi: 10.1161/circulationaha.118.037454 30798638

[ref44] Rodriguez C. J. Swett K. Agarwal S. K. Folsom A. R. Fox E. R. Loehr L. R. . (2014). Systolic blood pressure levels among adults with hypertension and incident cardiovascular events: the atherosclerosis risk in communities study. JAMA Intern. Med. 174, 1252–1261. 24935209 10.1001/jamainternmed.2014.2482 PMC4573449

[ref45] Rubini P. Subasini C. Katharine A. V. Kumaresan V. Kumar S. G. Nithya T. (2021). A cardiovascular disease prediction using machine learning algorithms. Ann. Rom. Soc. Cell Biol. 25, 904–912.

[ref46] Sen S. K. (2017). Predicting and diagnosing of heart disease using machine learning algorithms. Int. J. Eng. Comput.r Sci. 6, 21623–21631.

[ref47] Shantsila E. Lip G. Y. Shantsila A. Kurpas D. Beevers G. Gill P. S. . (2023). Antihypertensive treatment in people of very old age with frailty: time for a paradigm shift? J. Hypertens. 41, 1502–1510. doi: 10.1097/hjh.0000000000003495 37432893

[ref48] Shishehbori F. Awan Z. (2024). Enhancing cardiovascular disease risk prediction with machine learning models. arXiv:240117328. doi: 10.48550/arXiv.2401.17328

[ref49] Singh G. M. Danaei G. Farzadfar F. Stevens G. A. Woodward M. Wormser D. . (2013). The age-specific quantitative effects of metabolic risk factors on cardiovascular diseases and diabetes: a pooled analysis. PLoS One 8:e65174. doi: 10.1371/journal.pone.0065174 23935815 PMC3728292

[ref50] Solanki A. Barot M. P. (2019). Study of heart disease diagnosis by comparing various classification algorithms. International journal of engineering and advanced. Technology 8, 40–42.

[ref51] Terry A. L. Chiappa M. M. McAllister J. Woodwell D. A. Graber J. E. (2024). Plan and operations of the National Health and nutrition examination survey, August 2021-August 2023. Vital Health Stat., 1–21. doi: 10.15620/cdc/151927 38768042

[ref52] van Os H. J. Kanning J. P. Bonten T. N. Rakers M. M. Putter H. Numans M. E. . (2023). Cardiovascular risk prediction in men and women aged under 50 years using routine care data. J. Am. Heart Assoc. 12:e027011. 36942627 10.1161/JAHA.122.027011 PMC10122889

[ref53] Vinholt P. J. Hvas A.-M. Frederiksen H. Bathum L. Jørgensen M. K. Nybo M. (2016). Platelet count is associated with cardiovascular disease, cancer and mortality: a population-based cohort study. Thromb. Res. 148, 136–142. doi: 10.1016/j.thromres.2016.08.012 27586589

[ref54] Wallisch C. Agibetov A. Dunkler D. Haller M. Samwald M. Dorffner G. . (2021). The roles of predictors in cardiovascular risk models-a question of modeling culture? BMC Med. Res. Methodol. 21:284. doi: 10.1186/s12874-021-01487-4 34922459 PMC8684157

[ref55] Wang N. Li Y. Han S. Zhang Y. Yang J. Yin Z. . (2024). CFViSA: a comprehensive and free platform for visualization and statistics in omics-data. Comput. Biol. Med. 171:108206. doi: 10.1016/j.compbiomed.2024.108206 38430745

[ref56] Wang J. Xue Q. Zhang C. W. Wong K. K. L. Liu Z. (2024). Explainable coronary artery disease prediction model based on AutoGluon from AutoML framework. Front. Cardiovas. Med. 11:1360548. doi: 10.3389/fcvm.2024.1360548 PMC11246996 39011494

[ref57] World Health Organization. (2017) Health stats 2017. Available online at: https://www.who.int/health-topics/cardiovascular-diseases#tab=tab_1 (Accessed March 06, 2025).

[ref58] Yang M.-R. Wu Y.-W. (2023). A cross-validated feature selection (CVFS) approach for extracting the most parsimonious feature sets and discovering potential antimicrobial resistance (AMR) biomarkers. Comput. Struct. Biotechnol. J. 21, 769–779. doi: 10.1016/j.csbj.2022.12.046 36698972 PMC9842539

[ref59] Yazdani A. Varathan K. D. Chiam Y. K. Malik A. W. Wan Ahmad W. A. (2021). A novel approach for heart disease prediction using strength scores with significant predictors. BMC Med. Inform. Decis. Mak. 21:194. doi: 10.1186/s12911-021-01527-5 34154576 PMC8215833

[ref60] Zhao Y. Wood E. P. Mirin N. Cook S. H. Chunara R. (2021). Social determinants in machine learning cardiovascular disease prediction models: a systematic review. Am. J. Prev. Med. 61, 596–605. doi: 10.1186/s12911-021-01527-5 34544559

[ref61] Zhou L. Nutakor J. A. Larnyo E. Addai-Dansoh S. Cui Y. Gavu A. K. . (2024). Exploring socioeconomic status, lifestyle factors, and cardiometabolic disease outcomes in the United States: insights from a population-based cross-sectional study. BMC Public Health 24:2174. doi: 10.1186/s12889-024-19685-2 39134948 PMC11318151

