# Supplementary material for: Web-based cardiovascular disease risk prediction using machine learning
Source: Front Artif Intell. 2026 Feb 13;9:1690664. doi: 10.3389/frai.2026.1690664 (PMC12946134; doi:10.3389/frai.2026.1690664)
Supplement: Supplementary file 1 [file Data_Sheet_1.docx]

Supplementary Material

Web-Based Cardiovascular Disease Risk Prediction Using Machine Learning

Suraiya Akhter*, John H. Miller

*** Correspondence:** sakhter1@emporia.edu

# Supplementary Data

## Training dataset

The training dataset can be accessible at https://github.com/suraiya14/CVDRP.

## Testing dataset

The testing dataset can be accessible at https://github.com/suraiya14/CVDRP.

# Supplementary Tables

## Feature set before applying feature reduction algorithm

**Table S1.** List of features used for feature reduction analysis

| SMQ020 | PAD810Q | PAD790Q | PAD790U | PAD800 | PAD680 | SLD012 | SLD013 |
| --- | --- | --- | --- | --- | --- | --- | --- |
| DIQ010 | RIDAGEYR | RIAGENDR | RIDRETH3 | DMDEDUC2 | INDFMPIR | BMXBMI | BMXWAIST |
| BPXOSY1 | BPXODI1 | LBXTC | LBDHDD | LBXGH | LBXHSCRP | BPQ101D | BPQ020 |
| BPQ080 | RXQ033 | LBXSNASI | LBXWBCSI | LBXHGB | LBXPLTSI | LBXRDW |  |

## 2.2 Pearson correlation + Chi-squared test-reduced features

**Table S2.** List of features obtained from Pearson correlation + Chi-squared test

| PAD810Q | SLD012 | BMXBMI | LBXTC | LBXSNASI | LBXRDW | RIAGENDR |
| --- | --- | --- | --- | --- | --- | --- |
| PAD790Q | SLD013 | BMXWAIST | LBDHDD | LBXWBCSI | SMQ020 | RIDRETH3 |
| PAD800 | RIDAGEYR | BPXOSY1 | LBXGH | LBXHGB | PAD790U | PAD680 |
| INDFMPIR | BPXODI1 | LBXHSCRP | LBXPLTSI | DIQ010 |  |  |

**2.3 ADT-reduced features**

**Table S3.** List of features obtained from ADT

| RIDAGEYR | LBXTC | INDFMPIR | RXQ033 | LBXPLTSI |
| --- | --- | --- | --- | --- |
| BPQ020 | PAD800 | PAD790U | LBXSNASI | SLD013 |
| SMQ020 | PAD810Q | BPQ101D | PAD680 | BMXWAIST |

## 2.4 CVFE reduced features

In the following tables S4-S7, c, *e* and *p* indicate count of disjoint sub-parts, count of iterations and ratios of recurring iterations for the extraction of common features, respectively.

**2.4.1 Table S4.** List of features obtained from CVFE (*c* = 2, *e* = 10, *p* = 0.2)

| BPQ101D | SMQ020 | RIAGENDR | PAD810Q | PAD790U | PAD790Q | BPQ080 |
| --- | --- | --- | --- | --- | --- | --- |
| RIDAGEYR | INDFMPIR | LBXRDW | BMXBMI | LBXHSCRP | BPXODI1 |  |
| RXQ033 | SLD012 | DIQ010 | LBXWBCSI | RIDRETH3 | LBXPLTSI |  |
| LBXTC | PAD800 | SLD013 | PAD680 | DMDEDUC2 | BPXOSY1 |  |
| BPQ020 | LBXHGB | LBDHDD | LBXGH | LBXSNASI | BMXWAIST |  |

**2.4.2 Table S5.** List of features obtained from CVFE (*c* = 2, *e* = 10, *p* = 0.6)

| BPQ101D | SMQ020 | RIAGENDR | PAD810Q | PAD790U | PAD790Q | BPQ080 |
| --- | --- | --- | --- | --- | --- | --- |
| RIDAGEYR | INDFMPIR | LBXRDW | BMXBMI | LBXHSCRP | BPXODI1 |  |
| RXQ033 | SLD012 | DIQ010 | LBXWBCSI | RIDRETH3 | LBXPLTSI |  |
| LBXTC | PAD800 | SLD013 | PAD680 | DMDEDUC2 | BPXOSY1 |  |
| BPQ020 | LBXHGB | LBDHDD | LBXGH | LBXSNASI | BMXWAIST |  |

**2.4.3 Table S6.** List of features obtained from CVFE (*c* = 2, *e* = 5, *p* = 0.8)

| BPQ101D | BPQ020 | PAD800 | LBDHDD | LBXWBCSI | DMDEDUC2 | LBXPLTSI |
| --- | --- | --- | --- | --- | --- | --- |
| RIDAGEYR | SMQ020 | LBXHGB | PAD810Q | PAD680 | LBXSNASI | BPXOSY1 |
| RXQ033 | INDFMPIR | LBXRDW | PAD790U | LBXGH | PAD790Q | BMXWAIST |
| LBXTC | SLD012 | SLD013 | BMXBMI | LBXHSCRP | BPXODI1 | BPQ080 |

**2.4.4 Table S7.** List of features obtained from CVFE (*c* = 3, *e* = 5, *p* = 0.6)

| BPQ101D | RIAGENDR | PAD800 | LBDHDD | PAD680 | LBXSNASI | LBXPLTSI |
| --- | --- | --- | --- | --- | --- | --- |
| RIDAGEYR | SMQ020 | LBXHGB | PAD810Q | LBXGH | RIDRETH3 | BPXOSY1 |
| LBXTC | INDFMPIR | LBXRDW | BMXBMI | LBXHSCRP | PAD790Q | BMXWAIST |
| BPQ020 | SLD012 | SLD013 | LBXWBCSI | DMDEDUC2 | BPXODI1 | BPQ080 |

## 2.5 HFE reduced features

The lists of features obtained from the HFE method—using bin values of 5 and 10 to discretize each feature—are presented in Tables S7 and S8. In each table, the first 8, 16, and 23 features correspond to β values of 25, 50, and 75, respectively.

**2.5.1 Table S8.** List of features obtained from HFE with bin = 5

| BPQ101D | SMQ020 | LBXRDW | LBXGH | RIDRETH3 | BMXBMI | PAD810Q |
| --- | --- | --- | --- | --- | --- | --- |
| RIDAGEYR | LBXTC | BPXOSY1 | BMXWAIST | PAD790U | PAD790Q |  |
| BPQ020 | DIQ010 | INDFMPIR | LBXHGB | LBXSNASI | SLD013 |  |
| BPQ080 | RIAGENDR | LBXPLTSI | PAD680 | LBXWBCSI | PAD800 |  |
| RXQ033 | DMDEDUC2 | LBDHDD | SLD012 | BPXODI1 | LBXHSCRP |  |

**2.5.2 Table S9.** List of features obtained from HFE with bin = 10

| BPQ101D | LBXTC | LBXPLTSI | BMXWAIST | RIDRETH3 | PAD790Q | LBXHSCRP |
| --- | --- | --- | --- | --- | --- | --- |
| RIDAGEYR | RXQ033 | RIAGENDR | LBDHDD | PAD790U | SLD013 |  |
| BPQ020 | SMQ020 | DMDEDUC2 | PAD680 | LBXSNASI | BMXBMI |  |
| BPQ080 | BPXOSY1 | LBXRDW | LBXHGB | BPXODI1 | PAD800 |  |
| LBXGH | DIQ010 | INDFMPIR | SLD012 | LBXWBCSI | PAD810Q |  |

**3. Supplementary Figures**

**3.1 ADT**

**Figure S1 –** An alternating decision tree representation of the reduced feature set.

**
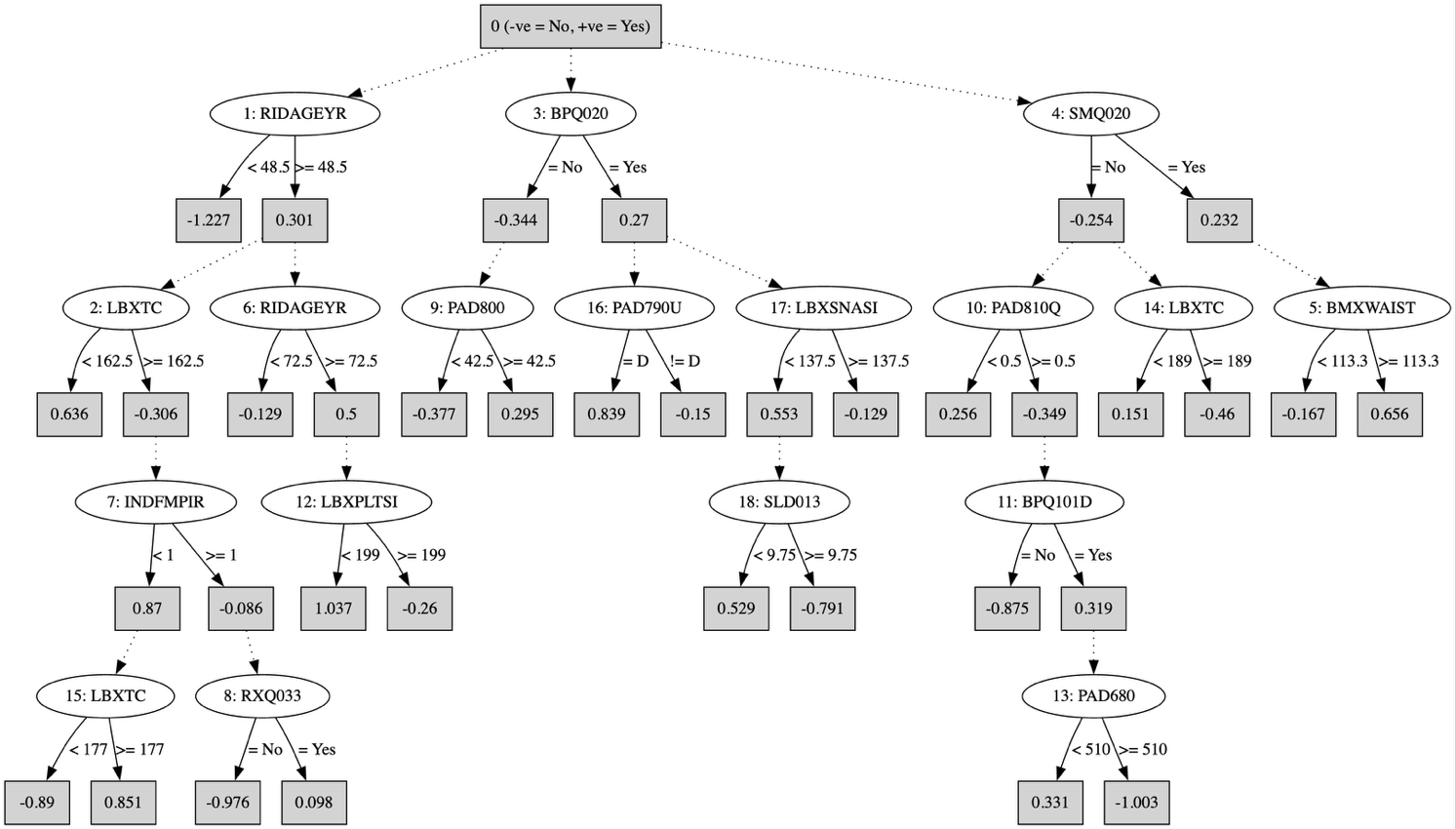
**

**3.2 Confusion matrices**

**Figure S2 –** Confusion matrices of the best-performing machine-learning models developed using the reduced feature sets. In these confusion matrices, “Positive” indicates participants with CVD, while “Negative” indicates participants without CVD.

| **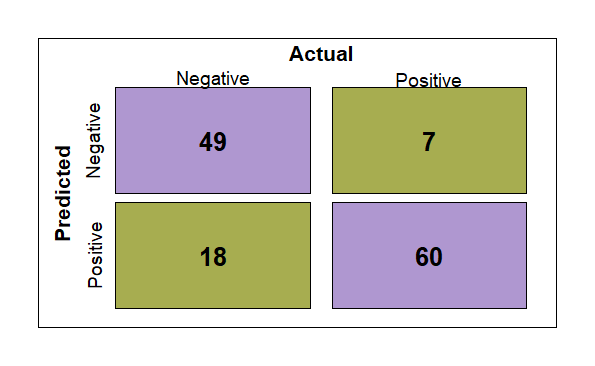**   1. **CVFE (*c* = 2, *e* = 10, *p* = 0.2) [RF]** | **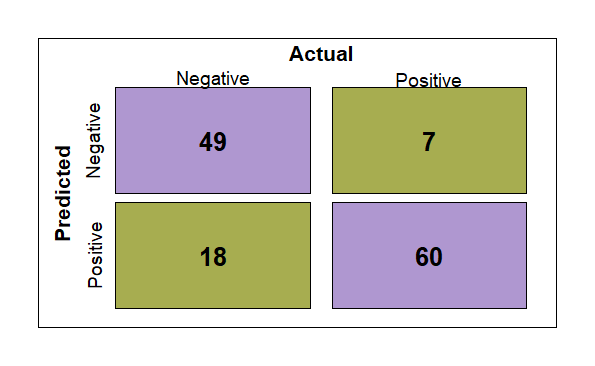**   1. **CVFE (*c* = 2, *e* = 10, *p* = 0.6) [RF]** | **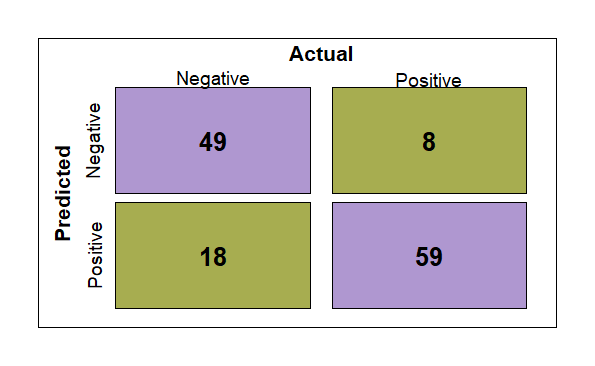**   1. **CVFE (*c* = 2, *e* = 5, *p* = 0.8) [RF]** | **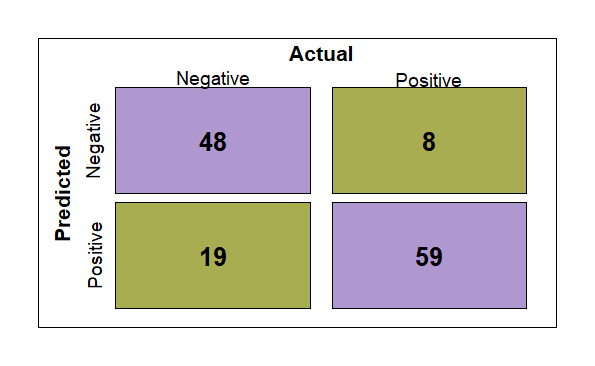**   1. **CVFE (*c* = 3, *e* = 5, *p* = 0.6) [RF]** |
| --- | --- | --- | --- |
| **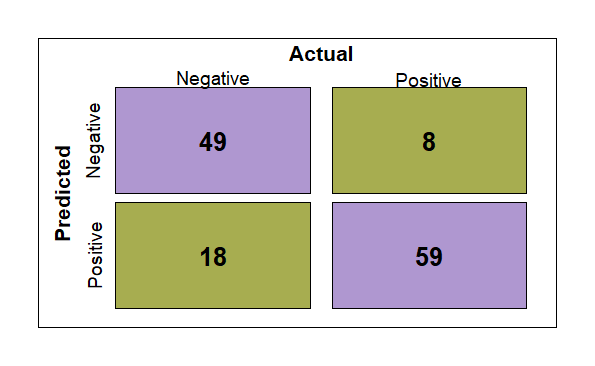**   1. **HFE (bin = 5, *β*** **= 25) [SVM]** | **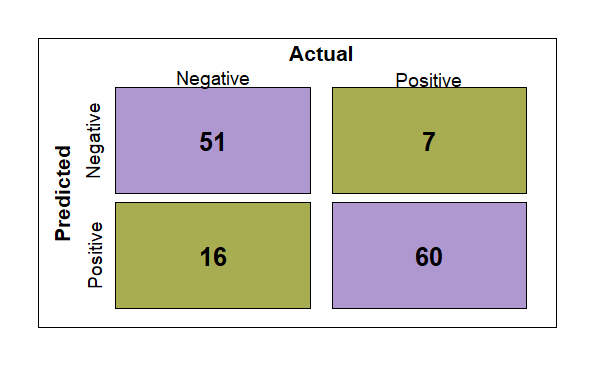**   1. **HFE (bin = 5, *β* = 50) [SVM]** | **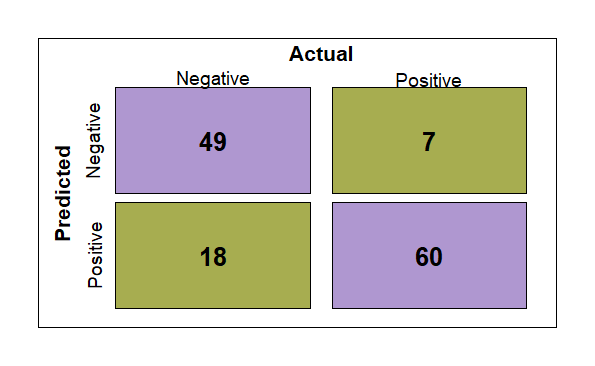**   1. **HFE (bin = 5, *β* = 75) [RF]** | **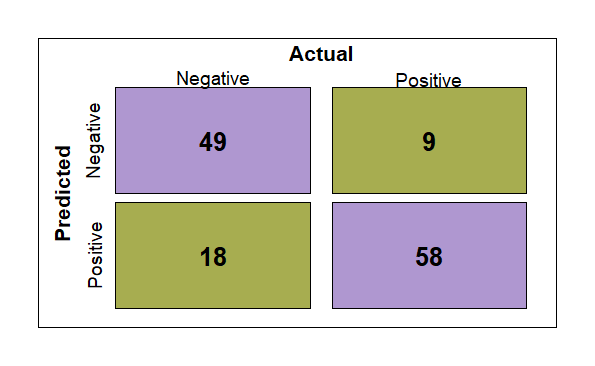**   1. **HFE (bin = 10, *β* = 25) [SVM]** |
| **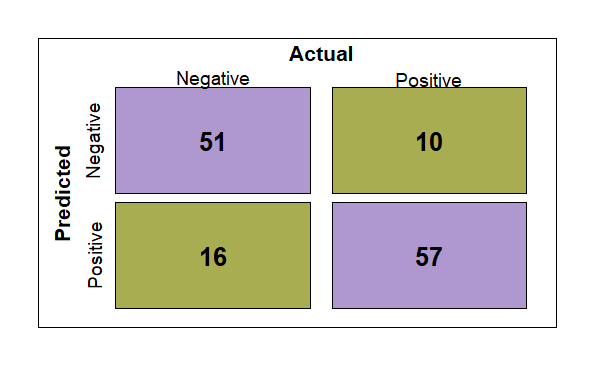**   1. **HFE (bin = 10, *β* = 50) [SVM]** | **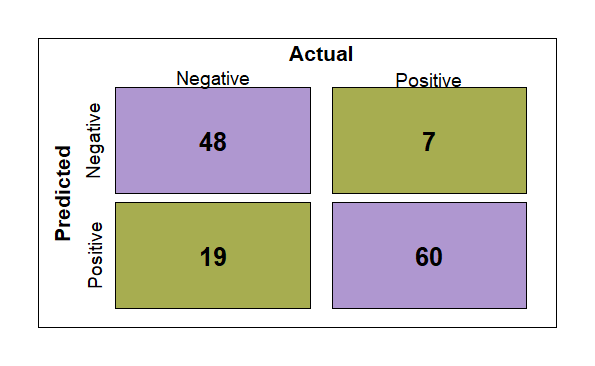**   1. **HFE (bin = 10, *β* = 75) [RF]** | **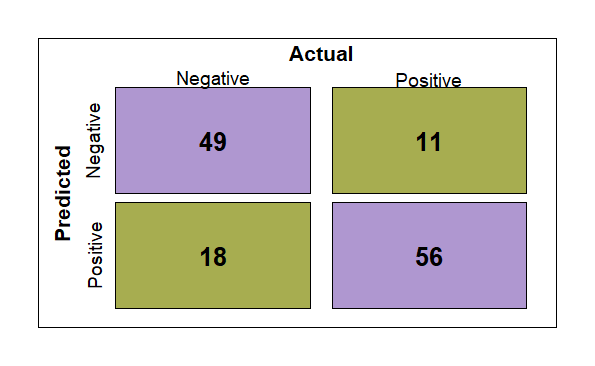**   1. **ADT [XGBoost]** | **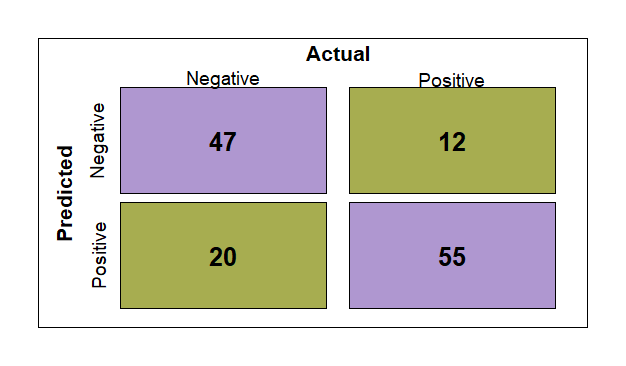**   1. **Pearson correlation + Chi-squared test [SVM]** |
